# Efficient synthesis of ethyl 2-(oxazolin-2-yl)alkanoates via ethoxycarbonylketene-induced electrophilic ring expansion of aziridines

**DOI:** 10.3762/bjoc.18.6

**Published:** 2022-01-05

**Authors:** Yelong Lei, Jiaxi Xu

**Affiliations:** 1 State Key Laboratory of Chemical Resource Engineering, Department of Organic Chemistry, College of Chemistry, Beijing University of Chemical Technology, Beijing 100029, People’s Republic of China

**Keywords:** aziridine, diazooxoester, diazo compound, ketene, oxazoline, ring expansion

## Abstract

Alkyl 2-diazo-3-oxoalkanoates generate alkoxycarbonylketenes, which undergo an electrophilic ring expansion with aziridines to afford alkyl 2-(oxazolin-2-yl)alkanoates in good to excellent yields under microwave heating. The method is a convenient and clean reaction without any activators and catalysts and can be also applied in the synthesis of 2-(oxazolin-2-yl)alkanamides and 1-(oxazolin-2-yl)alkylphosphonates.

## Introduction

**Figure 1:**
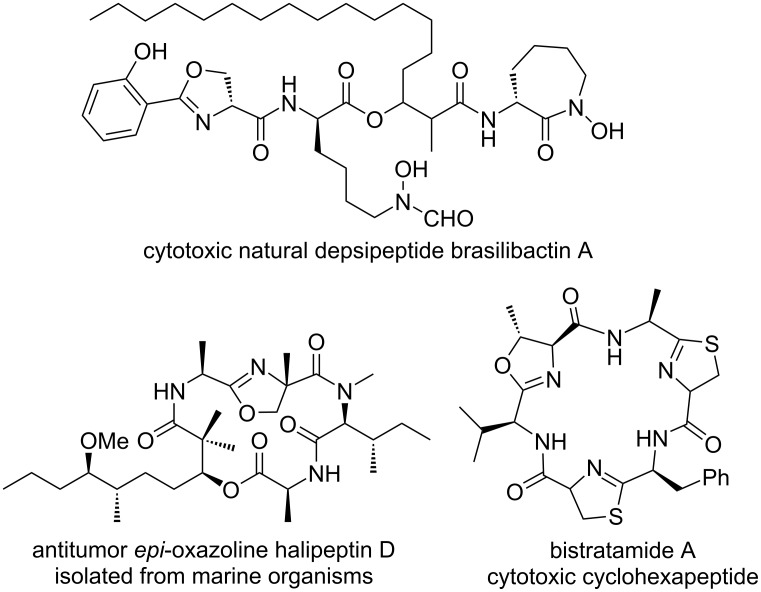
Oxazoline-containing bioactive natural products.

Oxazoline derivatives are an important class of nitrogen and oxygen-containing five-membered unsaturated heterocycles [1] and widely exist in some natural products [2] and pharmaceuticals [3], such as in the antitumor *epi*-oxazoline halipeptin D isolated from marine organisms [4], in the cytotoxic natural depsipeptide brasilibactin A [5], and cyclohexapeptide bistratamide A [6] (Figure 1). Oxazoline is also one of the crucial coordinating groups in symmetric and asymmetric ligands widely applied in various organic transformations [7]. Especially, bisoxazolines are a kind of widely applied chiral ligands in diverse transition metal-participating asymmetric catalysis [8–10].

**Scheme 1:**
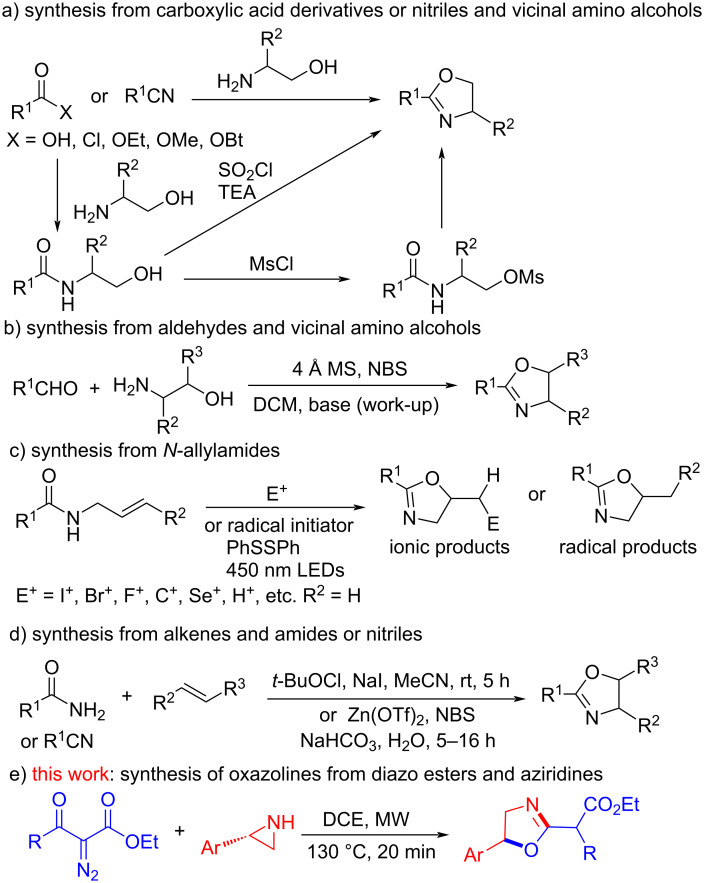
Synthetic methods of oxazoline derivatives.

**Scheme 2:**
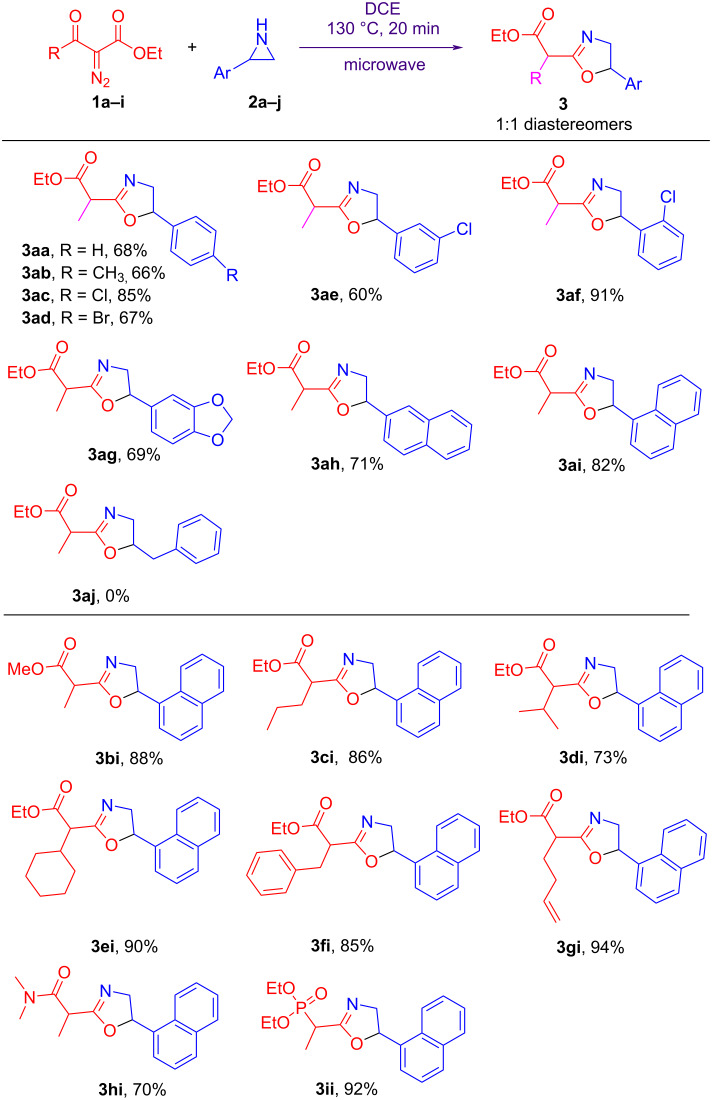
Scopes of aziridines and diazo esters.

**Scheme 3:**
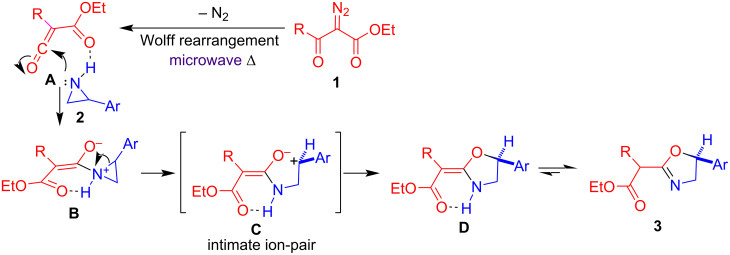
Proposed reaction mechanism.

**Scheme 4:**
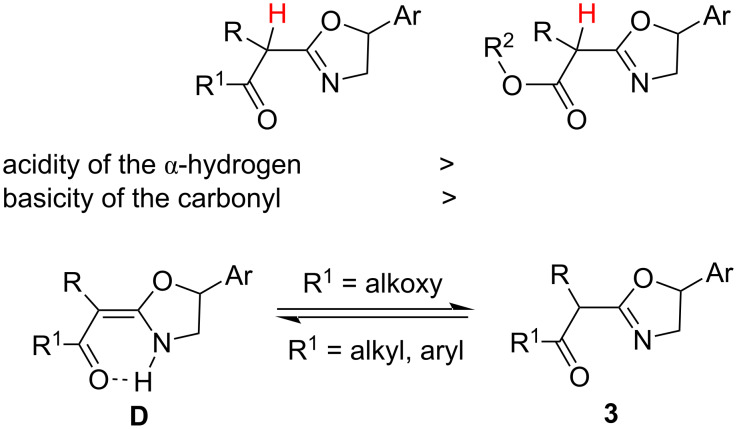
Direction of tautomerization.

Several methods have been developed for the efficient synthesis of oxazoline derivatives [11–12]. They mainly include (1) cyclization of 2-amidoethyl halides or sulfonates, which are prepared from carboxylic acid derivatives and vicinal amino alcohols [8–10] (Scheme 1a); (2) direct condensation of carboxylic acid derivatives or nitriles with vicinal amino alcohols [13–15] (Scheme 1a); (3) oxidative condensation of aldehydes with vicinal amino alcohols [16] (Scheme 1b); (4) cyclization of *N*-allylamides in the presence of electrophilic reagents or radical initiators or catalysts [17] (Scheme 1c); (5) direct synthesis from alkenes and amides or nitriles in the presence of electrophilic reagents [18–19] (Scheme 1d). Aziridines can be considered as the NCC structural fragment after ring-opening and have been applied in the synthesis of aziridine-imine-containing chiral tridentate ligands [20], 2-alkylideneoxazolidines [21], and *N*-vinylamides [22]. We envisioned that the reaction of ethoxycarbonylketenes and aziridines can be applied for the synthesis of ethyl 2-(oxazolin-2-yl)alkanoates. Herein, we present our convenient and clean synthesis of ethyl 2-(oxazolin-2-yl)alkanoates from 2-diazo-3-oxoalkanoates and 2-arylaziridines (Scheme 1e).

## Results and Discussion

The reaction of ethyl 2-diazo-3-oxobutanoate (**1a**) and 2-phenylaziridine (**2a**) was first selected as a model reaction to optimize the reaction conditions (Table 1). Diazo ester **1a** (0.36 mmol) and aziridine **2a** (0.3 mmol) in 1,2-dichloroethane (DCE, 1 mL) were heated at 110 °C for 30 min with microwave heating, affording the desired product **3aa** in 41% yield with remaining starting materials **1a** and **2a** (Table 1, entry 1). The reaction was further conducted at elevated temperatures 120 °C, 130 °C, and 140 °C, giving the product **3aa** in 64%, 68%, and 70%, respectively (Table 1, entries 2–4). Similar yields were obtained at 130 °C and 140 °C. The yield increased to 71% when the reaction time was shortened to 20 min (Table 1, entry 5). Further shortening the reaction time to 10 min resulted in the yield to drop to 66% (Table 1, entry 6). Changing the ratio of diazo ester **1a** and aziridine **2a** did not improve the yield (Table 1, entries 7–9). Solvent screening indicated that the same yield of 71% was obtained in toluene (Table 1, entry 13). However, lower yields were obtained in MeCN, THF, and 1,4-dioxane (Table 1, entries 10–12). Further optimizations in toluene were carried out. However, the yield was not further improved, even if the reaction was conducted at 140 °C and 150 °C (Table 1, entries 15–18). In each of these cases, a 1:1 mixture of diastereomeric product **3aa** was obtained. Finally, considering that the yield is slightly higher at 130 °C than that at 140 °C and DCE shows better solubility to all substrates than toluene, the optimal reaction conditions were selected as: diazo ester **1a** (0.36 mmol) and **2a** (0.3 mmol) in DCE (1 mL) were heated at 130 °C for 20 min with microwave heating.

**Table 1:** Optimization of reaction conditions^a^.

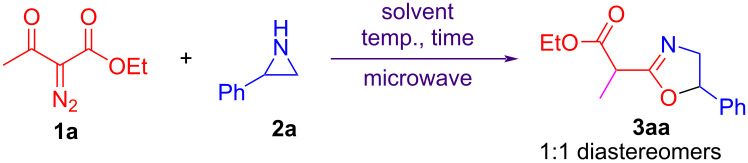					

Entry	Diazo ester **1a** (mmol)	Solvent	Temp. (°C)	Time (min)	Yield (%)^b^

1	0.36	DCE	110	30	41
2	0.36	DCE	120	30	64
3	0.36	DCE	130	30	68
4	0.36	DCE	140	30	70
5	0.36	DCE	130	20	71
6	0.36	DCE	130	10	66
7	0.30	DCE	130	20	63
8	0.45	DCE	130	20	53
9	0.60	DCE	130	20	62
10	0.36	MeCN	130	20	48
11	0.36	THF	120	20	10
12	0.36	1,4-dioxane	130	20	50
13	0.36	toluene	130	20	71
15	0.36	toluene	130	30	67
16	0.45	toluene	130	30	56
17	0.45	toluene	140	30	62
18	0.45	toluene	150	30	55

^a^All reactions were conducted with **1a** and **2a** (0.3 mmol) in solvent (1.0 mL) in a sealed 10 mL microwave tube and were stirred under microwave heating. ^b^The yield was determined by ^1^H NMR with 1,3,5-trimethoxybenzene as an internal standard.

With the optimal reaction conditions in hand, we evaluated the scopes and generalities of both diazo esters **1** and aziridines **2** (Scheme 2). Different 2-arylaziridines **2** were reacted with diazo ester **1a**, affording oxazolines **3aa–ai** in 60–91% yields. No obvious electronic effect was observed. Steric bulky 2-(2-chlorophenyl)aziridine (**2f**) gave the desired product **3af** in the highest yield of 91%. Steric 2-(naphth-1-yl)aziridine (**2i**) also exhibited a higher yield than 2-(naphth-2-yl)aziridine (**2h**). However, aliphatic aziridine 2-benzylaziridine (**2j**) did not give the corresponding product **3aj** when it reacted with diazo ester **1a** although **1a** decomposed under the reaction conditions. The reactions of different diazo esters **1** and aziridine **2i** were performed, generating the corresponding oxazolines **3bi–gi** in 73–94% yields. Ethyl 2-diazo-3-oxohept-6-enoate showed the highest activity, affording the desired product **3gi** in 94% yield. One diazo amide, 2-diazo-*N,N*-dimethyl-3-oxobutanamide (**1h**), was tested with aziridine **2i** as well, giving the desired product 2-(oxazolin-2-yl)propanamide **3hi** in 70% yield. Similarly, the reaction of diethyl 1-diazo-2-oxopropylphosphonate (**1i**) and aziridine **2i** gave rise to the corresponding product 1-(oxazolin-2-yl)alkylphosphonate **3ii** in 92% yield. In all cases, 1:1 mixtures of diastereomeric products **3** were obtained. Compared with previously reported methods, our current method is more convenient and low cost without any activators (such as thionyl chloride, sulfonyl chlorides, NBS, electrophiles, and radical initiators) and catalysts. The current synthetic strategy is a clean reaction and shows widely application in the preparation of 1-(oxazolin-2-yl)alkanoic acid derivatives and dialkyl 1-(oxazolin-2-yl)alkylphosphonates.

On the basis of the experimental results and previous reports [21–22], the reaction mechanism is rationalized as following (Scheme 3). Under microwave heating, diazo esters **1** undergo a Wolff rearrangement to generate ethoxycarbonylketenes **A** by loss of nitrogen. The nucleophilic attack of 2-arylaziridine **2** on the ketene moiety produces zwitterionic intermediates **B**, in which the aziridinium is opened to form the benzylic carbocation stabilized through the p–π conjugation. This stabilization is not possible with alkyl groups, explaining why 2-alkylaziridines did not generate the corresponding products. Intermediates **C** undergo an intramolecular nucleophilic attack to yield ethyl (oxazolidin-2-ylidene)alkanoates **D**, which further isomerize to more stable products, ethyl (oxazolin-2-yl)alkanoates **3**.

Interestingly, the reaction of α-diazo-β-diketones and 2-arylaziridines generated 2-(2-oxoalkylidene)oxazolidines [21], while the current reaction of alkyl α-diazo-β-oxoalkanoates and 2-arylaziridines gave alkyl (oxazolin-2-yl)alkanoates as products, showing different chemoselectivities. 2-Alkylideneoxazolidines and 2-alkyloxazolines are structural isomers and can possibly tautomerize each other. As a more electron-withdrawing group with more electron density on the carbonyl group, a ketone favors the conjugation of the double bond as well as the intramolecular hydrogen bond. Thus, the tautomerization favors to the left direction, forming D-form products, when α-diazo-β-diketones are as starting materials (R^1^ = alkyl and aryl), while it predominates to the right direction, generating 2-(alkoxycarbonyl)methyloxazolines as products, when alkyl α-diazo-β-oxoalkanoates are used in the reaction, showing specific chemoselectivities controlled by the electronic effect (Scheme 4).

## Conclusion

A new and efficient synthetic method for the synthesis of oxazolines has been developed with alkyl 2-diazo-3-oxoalkanoates and 2-arylaziridines as starting materials. Alkyl 2-diazo-3-oxoalkanoates first generate alkoxycarbonylketenes, which undergo an electrophilic ring expansion with aziridines to afford alkyl 2-(oxazolin-2-yl)alkanoates in good to excellent yields under microwave heating. The current method is an activator- and catalyst-free, and clean synthetic strategy and can be applied in the synthesis of 2-(oxazolin-2-yl)alkanamides and 1-(oxazolin-2-yl)alkylphosphonates as well, showing versatile application.

## Experimental

Unless otherwise noted, all materials were purchased from commercial suppliers without further purification. THF was refluxed over LiAlH_4_; DCE, MeCN, and 1,4-dioxane were refluxed over CaH_2_; toluene was refluxed over Na with benzophenone as an indicator, and all solvents were freshly distilled prior to use. Flash column chromatography was performed using silica gel (normal phase, 200–300 mesh) from Branch of Qingdao Haiyang Chemical. Petroleum ether (PE) used for column chromatography was the 60–90 °C fraction, and the removal of residual solvent was accomplished under rotovap. Reactions were monitored by thin-layer chromatography on silica gel GF254 coated 0.2 mm plates from Institute of Yantai Chemical Industry. Microwave-assisted reactions were conducted on a CEM discovery SP microwave reactor. The plates were visualized under UV light, as well as other TLC stains. ^1^H, ^13^C, and ^31^P NMR spectra were recorded on a Bruker 400 MHz spectrometer in CDCl_3_ with solvent peaks as internal standards, for ^31^P NMR, 85% H_3_PO_4_ as an external standard, and the chemical shifts (δ) are reported in parts per million (ppm). All coupling constants (*J*) in ^1^H NMR are absolute values given in hertz (Hz) with peaks labeled as singlet (s), broad singlet (brs), doublet (d), triplet (t), quartet (q), and multiplet (m). IR spectra (KBr pellets, v [cm^−1^]) were taken on a Bruker Tensor 27 FTIR spectrometer. HRMS measurements were carried out on a Waters Acquilty UPLC/Quattro Premier mass spectrometer.

Alkyl 2-diazo-3-oxoalkanoates **1** were synthesized by referring our previous procedure [22–23]. Their analytic data are identical to previously reported ones **1a**,**b** [23], **1c**,**d** [24], **1e**,**g** [25], **1f** [26], **1h** [27] and **1i** [28]. Aziridines **2** were prepared according to our previous method [21] and their analytic data are identical to previously reported ones **2a–f** [21] and **2g** [29].

### General procedure for the synthesis of ethyl 2-(oxazol-2-yl)alkanoates **3**

Diazo compound **1** (0.36 mmol) and aziridine **2** (0.30 mmol) were added to DCE (1.0 mL) in a sealed 10 mL microwave tube. The resulting solution was stirred at 130 °C for 20 min under microwave heating. After the reaction was completed, the resulting mixture was evaporated in vacuo. The crude residue was purified by silica gel column chromatography (PE/EA 2:1, v/v) to give product **3**.

## Supporting Information

File 1 Analytic data and copies of ^1^H, ^13^C, and ^31^P NMR spectra of compounds **3**.
