# Supplementary material for: Efficient synthesis of ethyl 2-(oxazolin-2-yl)alkanoates via ethoxycarbonylketene-induced electrophilic ring expansion of aziridines
Source: Beilstein J Org Chem. 2022 Jan 5;18:70–6. doi: 10.3762/bjoc.18.6 (PMC8744460; doi:10.3762/bjoc.18.6)
Supplement: File 1 — Analytic data and copies of 1H, 13C, and 31P NMR spectra of compounds 3. [file Beilstein_J_Org_Chem-18-70-s001.pdf]

BEILSTEIN JOURNAL OF ORGANIC CHEMISTRY

Supporting Information

for

Efficient synthesis of ethyl 2-(oxazolin-2-yl)alkanoates via ethoxycarbonylketene-induced electrophilic ring expansion of aziridines

Yelong Lei and Jiaxi Xu

Beilstein J. Org. Chem. **2022,** *18,* 70–76. doi:10.3762/bjoc.18.6

Analytic data and copies of ^{1}H, ^{13}C, and ^{31}P NMR spectra of compounds 3

License and Terms: This is a supporting information file under the terms of the Creative Commons Attribution License (https://creativecommons.org/licenses/by/4.0). Please note that the reuse, redistribution and reproduction in particular requires that the author(s) and source are credited and that individual graphics may be subject to special legal provisions.
The license is subject to the *Beilstein Journal of Organic Chemistry* terms and conditions: (https://www.beilstein-journals.org/bjoc/terms)

Contents

Analytic data of products **3**……………………………………………………..S2

Copies of ^{1}H, ^{13}C, and ^{31}P NMR spectra of products **3**………………….....S10

Analytic data of products **3**

Ethyl 2-(5-phenyl-4,5-dihydrooxazol-2-yl)propanoate (3aa)

Colorless oil (68 mg, 68%). A mixture of diastereoisomers (*syn*-isomer: *anti*-isomer = 1:1). ^{1}H NMR (400 MHz, $CDCl_3$) δ 7.39 - 7.28 (m, 10H), 5.52 (d, *J* = 8.0 Hz, 1H), 5.49 (d, *J* = 7.6 Hz, 1H), 4.31 (ddd, *J* = 10.4, 3.2, 1.2 Hz, 1H), 4.28 (ddd, *J* = 10.0, 3.2, 0.8 Hz, 1H), 4.21 (q, *J* = 7.1 Hz, 4H), 3.83 (ddd, *J* = 7.9, 4.1, 0.7 Hz, 1H), 3.79 (ddd, *J* = 7.8, 4.1, 0.7 Hz, 1H), 3.59 (q, *J* = 7.2 Hz, 1H), 3.54 (q, *J* = 7.2 Hz, 1H), 1.54 (d, *J* = 7.6 Hz, 3H), 1.52 (d, *J* = 7.6 Hz, 3H), 1.28 (t, *J* = 7.2 Hz, 3H), 1.27 (t, *J* = 7.2 Hz, 3H). ^{13}C NMR (101 MHz, $CDCl_3$) δ 170.8 (170.7), 165.43 (165.42), 141.0 (140.9), 128.8, 128.34 (128.30), 125.8 (125.7), 81.4 (81.3), 62.8 (62.7), 61.5, 40.13 (40.10), 14.5 (14.4), 14.1. IR (KBr) *v* 3349, 3032, 2984, 2942, 2878, 1739, 1672, 1591, 1454, 1312, 1238, 1202, 1089, 987, 955, 761, 700 cm^{-1}. HRMS-ESI (*m/z*): calcd for $C_{14}H_{18}NO_3^+$ $[M+H]^+$: 248.1281, found 248.1285.

Ethyl 2-(5-(4-methylphenyl)-4,5-dihydrooxazol-2-yl)propanoate (3ab)

Yellow oil (52 mg, 66%). A mixture of diastereoisomers (*syn*-isomer: *anti*-isomer = 1:1). ^{1}H NMR (400 MHz, $CDCl_3$) δ 7.23 - 7.13 (m, 8H), 5.48 (d, *J* = 8.0 Hz, 1H), 5.45 (d, *J* = 8.0 Hz, 1H), 4.32 - 4.24 (m, 2H), 4.20 (q, *J* = 7.2 Hz, 4H), 3.81 (ddd, *J* = 8.0, 1.2 Hz,, 1H), 3.78 (ddd, *J* = 8.4, 4.0, 0.8 Hz, 1H), 3.56 (q, *J* = 7.2 Hz, 1H), 3.52 (q, *J* = 7.2 Hz, 1H), 2.34 (s, 6H), 1.51 (t, *J* = 7.5 Hz, 6H), 1.28 (d, *J* = 7.1 Hz, 3H), 1.26 (d, *J* = 6.5 Hz, 3H). ^{13}C NMR (101 MHz, $CDCl_3$) δ 170.8 (170.7), 165.4, 138.2 (138.1), 137.9 (137.8), 129.4, 125.9 (125.8), 81.4 (81.3), 62.7 (62.6), 61.4, 40.1, 21.17, 14.5 (14.3), 14.1. HRMS-ESI (*m/z*): calcd for $C_{15}H_{20}NO_3^+$ $[M+H]^+$: 262.1438, found 262.1448.

Ethyl 2-(5-(4-chlorophenyl)-4,5-dihydrooxazol-2-yl)propanoate (3ac)

Colorless oil (72 mg, 85%). A mixture of diastereoisomers (*syn*-isomer: *anti*-isomer = 1:1). ^{1}H NMR (400 MHz, $CDCl_3$) δ 7.34 (d, *J* = 8.4 Hz, 4H), 7.24 (d, *J* = 8.4 Hz, 4H), 5.49 (d, *J* = 7.6 Hz, 1H), 5.47 (d, *J* = 7.6 Hz, 1H), 4.34 - 4.31 (m, 1H), 4.30 - 4.26 (m, 1H), 4.22 (q, *J* = 7.1 Hz, 4H), 3.78 (dd, *J* = 7.6, 2.0 Hz, 1H), 3.75 (dd, *J* = 7.6, 2.4 Hz, 1H), 3.59 (q, *J* = 7.6 Hz, 1H), 3.53 (q, *J* =

7.2 Hz, 1H), 1.53 (d, *J* = 7.5 Hz, 3H), 1.51 (d, *J* = 7.6 Hz, 3H), 1.28 (t, *J* = 7.2 Hz, 6H). ^{13}C NMR (101 MHz, $CDCl_3$) δ 170.7 (170.6), 165.3, 139.5, 139.4, 134.2 (134.1), 127.2 (127.1), 80.6 (80.5), 62.8 (62.7), 61.5, 40.1 (40.0), 14.5, 14.3 (14.1). HRMS-ESI (*m/z*): calcd for $C_{14}H_{17}ClNO_3^+$ $[M+H]^+$: 282.0891, found 282.0901.

Ethyl 2-(5-(4-bromophenyl)-4,5-dihydrooxazol-2-yl)propanoate (3ad)

Yellow oil (66 mg 67%). A mixture of diastereoisomers (*syn*-isomer: *anti*-isomer = 1:1). ^{1}H NMR (400 MHz, $CDCl_3$) *δ* 7.47 (d, *J* = 8.5 Hz, 4H), 7.16 (d, *J* = 8.4 Hz, 4H), 5.48 - 5.41 (m, 2H), 4.29 (ddd, *J* = 14.4, 2.8, 1.2 Hz, 1H), 4.26 (ddd, *J* = 10.0, 2.8, 1.2 Hz, 1H), 4.19 (q, *J* = 7.1 Hz, 4H), 3.76 (ddd, *J* = 7.6, 2.3, 0.8 Hz, 1H), 3.72 (ddd, *J* = 7.6, 2.4, 0.8 Hz, 1H), 3.56 (q, *J* = 7.4 Hz, 1H), 3.51 (q, *J* = 7.4 Hz, 1H), 1.51 (d, *J* = 7.3 Hz, 3H), 1.49 (d, *J* = 7.3 Hz, 3H), 1.26 (t, *J* = 7.1 Hz, 6H). ^{13}C NMR (101 MHz, $CDCl_3$) δ 170.7 (170.6), 165.3, 140.0 (139.9), 131.9, 127.5 (127.4), 122.3 (122.2), 80.6 (80.5), 62.8 (62.6), 61.5, 40.1 (40.0), 14.5 (14.3), 14.1. HRMS-ESI (*m/z*): calcd for $C_{14}H_{17}BrNO_3^+$ $[M+H]^+$: 326.0386, found 326.0395.

Ethyl 2-(5-(3-chlorophenyl)-4,5-dihydrooxazol-2-yl)propanoate (3ae)

Yellow oil (50 mg 60%). A mixture of diastereoisomers (*syn*-isomer: *anti*-isomer = 1:1). ^{1}H NMR (400 MHz, $CDCl_3$) *δ* 7.33 - 7.27 (m, 6H), 7.19 - 7.14 (m, 2H), 5.48 (d, *J* = 7.6 Hz, 1H), 5.48 (d, *J* = 8.0 Hz, 1H), 4.34 - 4.30 (m, 1H), 4.30 - 4.26 (m, 1H), 4.23 (q, *J* = 7.1 Hz, 4H), 3.79 (dd, *J* = 7.7, 2.3 Hz, 1H), 3.75 (dd, *J* = 7.5, 2.1 Hz, 1H), 3.59 (q, *J* = 7.3 Hz, 1H), 3.53 (q, *J* = 7.2 Hz, 1H), 1.53 (d, *J* = 7.3 Hz, 3H), 1.51 (d, *J* = 7.3 Hz, 3H), 1.28 (t, *J* =7.1 Hz, 6H). ^{13}C NMR (101 MHz, $CDCl_3$) *δ* 170.7 (170.6), 165.34, 143.1 (143.0), 134.7, 130.1, 128.44 (128.40), 125.79 (125.76), 123.8 (123.7), 80.4 (80.3), 62.8 (62.7), 61.6, 40.10 (40.06), 14.5 (14.3), 14.1. HRMS-ESI (*m/z*): calcd for $C_{14}H_{17}ClNO_3^+$ $[M+H]^+$: 282.0819, found 282.0895.

Ethyl 2-(5-(2-chlorophenyl)-4,5-dihydrooxazol-2-yl)propanoate (3af)

Yellow oil (77 mg 91%). A mixture of diastereoisomers (*syn*-isomer: *anti*-isomer = 1:1). ^{1}H NMR (400 MHz, $CDCl_3$) δ 7.46 - 7.41 (m, 2H), 7.40 - 7.33 (m, 2H), 7.34 - 7.19 (m, 4H), 5.87 - 5.81 (m, 2H), 4.48 - 4.44 (m, 1H), 4.44 -

4.40 (m, 1H), 4.25 (q, *J* = 7.1 Hz, 4H), 3.70 (dd, *J* = 7.9, 1.0 Hz, 1H), 3.67 (dd, *J* = 7.9, 1.0 Hz, 1H), 3.64 (q, *J* = 7.6 Hz, 1H), 3.58 (q, *J* = 7.6 Hz, 1H), 1.57 (d, *J* = 7.6 Hz, 3H), 1.54 (d, *J* = 7.6 Hz, 3H), 1.31 (t, *J* = 7.1 Hz, 6H). ^{13}C NMR (101 MHz, $CDCl_3$) δ 170.8 (170.7), 165.1, 139.0 (138.9), 131.1 (131.0), 129.5 (129.4), 129.01 (128.98), 127.14 (127.09), 125.9, 78.2 (78.1), 62.2 (62.1), 61.5, 40.2 (40.1), 14.5 (14.4), 14.1. HRMS-ESI (*m/z*): calcd for $C_{14}H_{17}ClNO_3^+$ [M+H]$^+$: 282.0819, found 282.0898.

Ethyl 2-(5-(benzo[*d*][1,3]dioxol-5-yl)-4,5-dihydrooxazol-2-yl)propanoate (3ag)

Yellow oil (60 mg, 69%). A mixture of diastereoisomers (*syn*-isomer: *anti*-isomer = 1:1). ^1H NMR (400 MHz, $CDCl_3$) δ 6.86 – 6.72 (m, 6H), 5.96 (s, 4H), 5.44 (d, *J* = 8.0 Hz, 1H), 5.41 (d, *J* = 8.0 Hz, 1H), 4.29 – 4.22 (m, 2H), 4.21 (q, *J* = 7.1 Hz, 4H), 3.79 (ddd, *J* = 7.7, 3.9, 0.9 Hz, 1H), 3.76 (ddd, *J* = 7.7, 4.0, 0.9 Hz, 1H), 3.57 (q, *J* = 7.3 Hz, 1H), 3.51 (q, *J* = 7.3 Hz, 1H), 1.52 (d, *J* = 7.3 Hz, 3H), 1.50 (d, *J* = 7.3 Hz, 3H), 1.28 (t, *J* = 7.1 Hz, 6H). ^{13}C NMR (101 MHz, $CDCl_3$) δ 170.8 (170.7), 165.4, 148.2, 147.70 (147.66), 134.8 (134.7), 119.8 (119.6), 108.2, 106.2, 101.2, 81.4 (81.3), 62.7 (62.5), 61.5, 40.11 (40.08), 14.5 (14.3), 14.1. HRMS-ESI (*m/z*): calcd for $C_{15}H_{18}NO_5^+$ [M+H]$^+$: 292.1179, found 292.1187.

Ethyl 2-(5-(naphthalen-2-yl)-4,5-dihydrooxazol-2-yl)propanoate (3ah)

Yellow oil (63 mg, 71%). A mixture of diastereoisomers (*syn*-isomer: *anti*-isomer = 1:1). ^1H NMR (400 MHz, $CDCl_3$) δ 7.92 – 7.83 (m, 6H), 7.77 (s, 2H), 7.55 – 7.50 (m, 4H), 7.44 (d, *J* = 8.4 Hz, 2H), 5.71 (d, *J* = 7.6 Hz, 1H), 5.69 (d, *J* = 7.6 Hz, 1H), 4.43 – 4.39 (m, 1H), 4.39 – 4.35 (m, 1H), 4.26 (q, *J* = 7.2 Hz, 4H), 3.95 (dd, *J* = 8.0, 4.0 Hz, 1H), 3.91 (dd, *J* = 8.0, 4.0 Hz, 1H), 3.66 (q, *J* = 7.2 Hz, 1H), 3.61 (q, *J* = 7.2 Hz, 1H), 1.60 (d, *J* = 8.0 Hz, 3H), 1.58 (d, *J* = 8.0 Hz, 3H), 1.31 (t, *J* = 7.2 Hz, 6H). ^{13}C NMR (101 MHz, $CDCl_3$) δ 170.8 (170.7), 165.6 (165.5), 138.1 (138.0), 133.21 (133.20), 133.1, 128.97 (128.95), 127.97 (127.96), 127.8, 126.49 (126.47), 126.33 (126.31), 125.1 (125.0), 123.30 (123.25),

81.6 (81.5), 62.7 (62.6), 61.5, 40.19 (40.17), 14.5 (14.4), 14.2. HRMS-ESI (*m/z*): calcd for $C_{18}H_{20}NO_3^+$ $[M+H]^+$: 298.1438, found 298.1440.

Ethyl 2-(5-(naphthalen-1-yl)-4,5-dihydrooxazol-2-yl)propanoate (3ai)

Yellow oil (73 mg, 82%). A mixture of diastereoisomers (*syn*-isomer: *anti*-isomer = 1:1). ^{1}H NMR (400 MHz, $CDCl_3$) δ 7.92 - 7.88 (m, 2H), 7.81 (d, *J* = 8.0 Hz, 2H), 7.77 - 7.73 (m, 2H), 7.60 - 7.50 (m, 6H), 7.50 - 7.45 (m, 2H), 6.22 (dd, *J* = 8.0, 2.4 Hz, 1H), 6.20 (dd, *J* = 8.4, 2.4 Hz, 1H), 4.58 - 4.54 (m, 1H), 4.54 - 4.50 (m, 1H), 4.27 (q, *J* = 7.1 Hz, 4H), 3.84 (ddd, *J* = 8.4, 3.2, 0.8 Hz, 1H), 3.81 (ddd, *J* = 8.0, 3.2, 0.8 Hz, 1H), 3.69 (q, *J* = 7.2 Hz, 1H), 3.63 (q, *J* = 7.2 Hz, 1H), 1.61 (d, *J* = 7.2 Hz, 3H), 1.60 (d, *J* = 7.2 Hz, 3H), 1.31 (t, *J* = 7.2 Hz, 6H). ^{13}C NMR (101 MHz, $CDCl_3$) δ 170.83 (170.80), 165.52 (165.50), 136.4 (136.3), 133.85 (133.84), 129.59 (129.54), 129.1, 128.5 (128.4), 126.45 (126.43), 125.86 (125.84), 125.44 (125.39), 122.68 (122.67), 122.0, 79.21 (79.17), 62.4 (62.3), 61.5, 40.29 (40.25), 14.58 (14.55), 14.2. HRMS-ESI (*m/z*): calcd for $C_{18}H_{20}NO_3^+$ $[M+H]^+$: 298.1438, found 298.1441.

Methyl 2-(5-(naphthalen-1-yl)-4,5-dihydrooxazol-2-yl)propanoate (3bi)

Yellow oil (74 mg, 88%). A mixture of diastereoisomers (*syn*-isomer: *anti*-isomer = 1:1). ^{1}H NMR (400 MHz, $CDCl_3$) δ 7.92 - 7.89 (m, 2H), 7.82 (d, *J* = 8.2 Hz, 2H), 7.79 - 7.75 (m, 2H), 7.56 - 7.50 (m, 6H), 7.47 (dt, *J* = 2.2, 7.6 Hz, 2H), 6.22 (d, *J* = 8.4 Hz, 1H), 6.19 (d, *J* = 8.4 Hz, 1H), 4.57 - 4.54 (m, 1H), 4.52 (ddd, *J* = 10.5, 2.6, 1.1 Hz, 1H), 3.85 (ddd, *J* = 8.4, 3.2, 1.2 Hz, 1H), 3.83 - 3.81 (m, 1H), 3.81 (s, 6H), 3.73 - 3.70 (m, 1H), 3.70 - 3.65 (m, 1H), 1.62 (d, *J* = 7.4 Hz, 3H), 1.60 (d, *J* = 7.4 Hz, 3H). ^{13}C NMR (101 MHz, $CDCl_3$) δ 171.2, 165.4, 136.3 (136.2), 133.9, 129.59 (129.55), 129.1, 128.6 (128.5), 126.5, 125.9, 125.5 (125.4), 122.7, 122.11 (122.09), 79.4 (79.3), 62.3 (62.2), 52.59 (52.57), 40.1, 14.55 (14.53). HRMS-ESI (*m/z*): calcd for $C_{17}H_{18}NO_3^+$ $[M+H]^+$: 284.1281, found 284.1291.

Ethyl 2-(5-(naphthalen-1-yl)-4,5-dihydrooxazol-2-yl)pentanoate (3ci)

Yellow oil (84 mg, 86%). A mixture of diastereoisomers (*syn*-isomer: *anti*-isomer = 1:1). ^{1}H NMR (400 MHz, $CDCl_3$) δ 7.92 - 7.88 (m, 2H), 7.81 (d, *J* = 8.2

Hz, 2H), 7.77 - 7.72 (m, 2H), 7.59 - 7.55 (m, 2H), 7.55 - 7.50 (m, 4H), 7.47 (t, *J* = 7.2 Hz, 2H), 6.21 (dd, *J* = 8.0, 2.4 Hz, 1H), 6.18 (dd, *J* = 8.0, 2.4 Hz, 1H), 4.56 (ddd, *J* = 10.4, 1.6, 1.0 Hz, 1H), 4.52 (ddd, *J* = 10.5, 1.6, 1.0 Hz, 1H), 4.27 (d, *J* = 7.2 Hz, 4H), 3.84 (d, *J* = 8.4 Hz, 1H), 3.81 (d, *J* = 8.4 Hz, 1H), 3.60 (t, *J* = 7.6 Hz, 1H), 3.55 (t, *J* = 7.6 Hz, 1H), 2.12 - 1.99 (m, 4H), 1.493 (hexet, *J* = 7.6 Hz, 2H), 1.489 (hexet, *J* = 7.6 Hz, 2H), 1.313 (t, *J* = 7.2 Hz, 3H), 1.310 (t, *J* = 7.2 Hz, 3H), 1.01 (d, *J* = 7.2 Hz, 6H). ^{13}C NMR (101 MHz, $CDCl_3$) δ 170.3, 164.8 (164.7), 136.44 (136.40), 133.9, 129.6, 129.1, 128.5, 126.4, 125.9, 125.4, 122.7, 122.0 (121.9), 79.1 (79.0), 62.34 (62.27), 61.4, 45.84 (45.76), 31.5 (31.4), 20.7, 14.2, 13.8. HRMS-ESI (*m/z*): calcd for $C_{20}H_{24}NO_3^+$ $[M+H]^+$: 326.1751, found 326.1754.

Ethyl 3-methyl-2-(5-(naphthalen-1-yl)-4,5-dihydrooxazol-2-yl)butanoate (3di)

Yellow oil (74 mg, 73%). A mixture of diastereoisomers (*syn*-isomer: *anti*-isomer = 1:1). ^{1}H NMR (400 MHz, $CDCl_3$) *δ* 7.91 - 7.88 (m, 2H), 7.81 (d, *J* = 8.0 Hz, 2H), 7.77 - 7.71 (m, 2H), 7.61 - 7.55 (m, 2H), 7.55 - 7.49 (m, 4H), 7.47 (t, *J* = 8.0 Hz, 2H), 6.21 (dd, *J* = 6.6, 3.6 Hz, 1H), 6.18 (dd, *J* = 6.7, 3.7 Hz, 1H), 4.57 (d, *J* = 10.6 Hz, 1H), 4.54 (d, *J* = 10.6 Hz, 1H), 4.26 (q, *J* = 7.2 Hz, 4H), 3.84 (t, *J* = 9.2 Hz, 1H), 3.81 (t, *J* = 9.2 Hz, 1H), 3.37 (d, *J* = 8.8 Hz, 1H), 3.33 (d, *J* = 8.8 Hz, 1H), 2.63 - 2.49 (m, 2H), 1.32 (t, *J* = 7.1 Hz, 3H), 1.31 (t, *J* = 7.1 Hz, 3H), 1.14 (d, *J* = 6.4 Hz, 3H), 1.13 (d, *J* = 6.8 Hz, 3H), 1.10 (d, *J* = 6.8 Hz, 6H). ^{13}C NMR (101 MHz, $CDCl_3$) *δ* 169.7, 164.2 (164.0), 136.5 (136.4), 133.8, 129.6 (129.5), 129.1, 128.44 (128.38), 126.43 (126.39), 125.85 (125.83), 125.44 (125.43), 122.8 (122.7), 121.9, 78.9 (78.8), 62.25 (62.23), 61.3 (61.2), 53.3, 53.0, 29.13 (29.06), 20.7 (20.6), 14.2. HRMS-ESI (*m/z*): calcd for $C_{20}H_{24}NO_3^+$ $[M+H]^+$: 326.1751, found 326.1756.

Ethyl 2-cyclohexyl-2-(5-(naphthalen-1-yl)-4,5-dihydrooxazol-2-yl)acetate (3ei)

Yellow oil (98 mg, 90%). A mixture of diastereoisomers (*syn*-isomer: *anti*-isomer = 1:1). ^{1}H NMR (400 MHz, $CDCl_3$) *δ* 7.92 - 7.88 (m, 2H), 7.81 (d, *J* = 8.2 Hz, 2H), 7.76 - 7.70 (m, 2H), 7.62 - 7.58 (d, *J* = 7.6 Hz, 2H), 7.56 - 7.50

(m, 4H), 7.46 (t, *J* = 7.6 Hz, 2H), 6.21 (d, *J* = 8.4 Hz, 1H), 6.17 (d, *J* = 8.4 Hz, 1H), 4.56 (d, *J* = 10.4 Hz, 1H), 4.53 (d, *J* = 10.4 Hz, 1H), 4.25 (q, *J* = 6.8 Hz, 4H), 3.83 (dd, *J* = 14.0, 8.4 Hz, 1H), 3.80 (dd, *J* = 14.0, 8.4 Hz, 1H), 3.41 (d, *J* = 9.3 Hz, 1H), 3.36 (d, *J* = 9.2 Hz, 1H), 2.33 - 2.17 (m, 2H), 1.96 - 1.66 (m, 12H), 1.41 - 1.34 (m, 2H), 1.31 (t, *J* = 7.1 Hz, 3H), 1.30 (t, *J* = 7.1 Hz, 3H), 1.25 - 1.12 (m, 6H). ^{13}C NMR (101 MHz, $CDCl_3$) δ 169.69 (169.65), 164.0 (163.8), 136.5 (136.4), 133.8, 129.6 (129.5), 129.1, 128.43 (128.36), 126.41 (126.38), 125.84 (125.82), 125.45 (125.38), 122.8 (122.7), 121.92, 79.0 (78.8), 62.3 (62.2), 61.3 (61.2), 52.5, 52.3, 38.3 (38.1), 31.06 (30.95), 30.9, 26.2 (26.1), 26.0, 14.3. HRMS-ESI (*m/z*): calcd for $C_{23}H_{28}NO_3^+$ $[M+H]^+$: 366.2064, found 366.2077.

Ethyl 2-(5-(naphthalen-1-yl)-4,5-dihydrooxazol-2-yl)-3-phenylpropanoate (3fi)

Yellow oil (95 mg, 85%). A mixture of diastereoisomers (*syn*-isomer: *anti*-isomer = 1:1). ^{1}H NMR (400 MHz, $CDCl_3$) δ 7.92 - 7.86 (m, 2H), 7.83 - 7.77 (m, 2H), 7.75 - 7.67 (m, 2H), 7.54 - 7.48 (m, 4H), 7.46 - 7.35 (m, 4H), 7.38 - 7.24 (m, 10H), 6.17 (dd, *J* = 8.0, 1.6 Hz, 1H), 6.14 (dd, *J* = 8.0, 1.6 Hz, 1H), 4.51 (dd, *J* = 13.6, 10.4 Hz, 1H), 4.47 (dd, *J* = 13.6, 10.4 Hz, 1H), 4.29 - 4.21 (m, 1H), 4.22 (q, *J* = 7.2 Hz, 2H), 4.19 (q, *J* = 7.2 Hz, 2H), 3.93 - 3.88 (m, 1H), 3.88 - 3.84 (m, 1H), 3.80 (dd, *J* = 14.8, 6.4 Hz, 1H), 3.76 (dd, *J* = 15.6, 7.2 Hz, 1H), 3.42 - 3.31 (m, 3H), 1.23 (t, *J* = 7.2 Hz, 3H), 1.20 (t, *J* = 7.2 Hz, 3H). ^{13}C NMR (101 MHz, $CDCl_3$) δ 169.62 (169.58), 164.2 (164.0), 138.06 (138.02), 136.23 (136.16), 133.8, 129.6 (129.5), 129.1 (129.0), 128.9, 128.56 (128.51), 128.4, 127.9, 126.78 (126.74), 126.45 (126.43), 125.8, 125.45 (125.42), 122.68 (122.64), 122.2, 122.0, 79.1, 62.4 (62.3), 61.61 (61.57), 47.8 (47.5), 35.5 (35.4), 14.11 (14.09). HRMS-ESI (*m/z*): calcd for $C_{24}H_{24}NO_3^+$ $[M+H]^+$: 374.1751, found 374.1754.

Ethyl 2-(5-(naphthalen-1-yl)-4,5-dihydrooxazol-2-yl)hex-5-enoate (3gi)

Yellow oil (95 mg, 94%). A mixture of diastereoisomers (*syn*-isomer: *anti*-isomer = 1:1). ^{1}H NMR (400 MHz, $CDCl_3$) δ 7.93 - 7.88 (m, 2H), 7.81 (d, *J* = 8.0

Hz, 2H), 7.77 – 7.73 (m, 2H), 7.59 – 7.52 (m, 6H), 7.52 – 7.45 (m, 2H), 6.23 – 6.17 (m, 2H), 5.90 – 5.79 (m, 2H), 5.14 – 5.08 (m, 2H), 5.08 – 5.03 (m, 2H), 4.56 (ddd, *J* = 10.4, 3.6, 0.9 Hz, 1H), 4.53 (ddd, *J* = 10.4, 3.6, 0.9 Hz, 1H), 4.27 (q, *J* = 7.1 Hz, 4H), 3.85 (dd, *J* = 8.0, 0.8 Hz, 1H), 3.82 (dd, *J* = 8.0, 0.8 Hz, 1H), 3.61 (t, *J* = 7.2 Hz, 1H), 3.58 (t, *J* = 7.2 Hz, 1H), 2.26 – 2.16 (m, 8H) , 1.316 (t, *J* = 7.2 Hz, 3H), 1.314 (t, *J* = 7.2 Hz, 3H). ^{13}C NMR (101 MHz, $CDCl_3$) δ 170.1, 164.5 (164.4), 137.0, 136.38 (136.34), 133.8, 129.6, 129.1, 128.5, 126.4, 125.9, 125.4, 122.7, 122.0 (121.9), 116.0, 79.1 (79.0), 62.34 (62.27), 61.5, 45.2 (45.1), 31.4, 28.6 (28.5), 14.2. HRMS-ESI (*m/z*): calcd for $C_{21}H_{24}NO_3^+$ [M+H]$^+$: 338.1751, found 338.1755.

_N,N_-Dimethyl-2-(5-(naphthalen-1-yl)-4,5-dihydrooxazol-2-yl)propanamide (3hi)

Yellow oil (62 mg, 70%). A mixture of diastereoisomers (*syn*-isomer: *anti*-isomer = 1:1). ^{1}H NMR (400 MHz, $CDCl_3$) *δ* 7.92 – 7.88 (m, 2H), 7.81 (d, *J* = 7.6 Hz, 2H), 7.74 – 7.70 (m, 2H), 7.54 – 7.43 (m, 8H), 6.21 (d, *J* = 8.4 Hz, 1H), 6.18 (d, *J* = 8.4 Hz, 1H), 4.57 – 4.52 (m, 1H), 4.52 – 4.47 (m, 1H), 3.90 (q, *J* = 6.9 Hz, 2H), 3.83 (ddd, *J* = 8.0, 2.2, 1.1 Hz, 1H), 3.79 (ddd, *J* = 8.0, 2.2, 1.1 Hz, 1H), 3.16 (s, 3H), 3.14 (s, 3H), 3.05 (s, 3H), 3.04 (s, 3H), 1.59 (d, *J* = 7.2 Hz, 3H), 1.58 (d, *J* = 6.8 Hz, 3H). ^{13}C NMR (101 MHz, $CDCl_3$) δ 170.0 (169.9), 166.2 (166.1), 136.5, 133.9 (133.8), 129.5, 129.1, 128.5 (128.4), 126.44 (126.41), 125.9 (125.8), 125.5 (125.4), 122.7, 121.9 (121.7), 79.0, 62.21 (62.16), 37.62 (37.60), 37.34 (37.28), 36.13 (36.09), 14.9 (14.8). HRMS-ESI (*m/z*): calcd for $C_{18}H_{21}N_2O_2^+$ [M+H]$^+$: 297.1598, found 297.1609.

Diethyl (1-(5-(naphthalen-1-yl)-4,5-dihydrooxazol-2-yl)ethyl)phosphonate (3ii)

Yellow oil (100 mg, 92%). A mixture of diastereoisomers (*syn*-isomer: *anti*-isomer = 1:1). ^{1}H NMR (400 MHz, $CDCl_3$) *δ* 7.92 – 7.85 (m, 2H), 7.80 (d, *J* = 8.2 Hz, 2H), 7.75 – 7.70 (m, 4H), 7.55 – 7.45 (m, 6H), 6.23 (d, *J* = 8.4 Hz, 1H), 6.20 (d, *J* = 8.4 Hz, 1H), 4.59 – 4.53 (m, 1H), 4.53 – 4.46 (m, 1H), 4.25 – 4.15 (m, 8H), 3.81 (dd, *J* = 9.2, 4.4 Hz, 1H), 3.77 (dd, *J* = 8.5, 5.1 Hz, 1H),

3.28 - 3.21 (m, 1H), 3.21 - 3.13 (m, 1H), 1.63 (dd, *J* = 7.3, 6.0 Hz, 3H), 1.59 (dd, *J* = 7.2, 6.2 Hz, 3H), 1.33 (t, *J* = 7.2 Hz, 6H). 1.31 (t, *J* = 7.2 Hz, 6H). ^{13}C NMR (101 MHz, $CDCl_3$) δ 164.7 (d, *J* = 6.4 Hz) (164.6 (d, *J* = 5.9 Hz)), 136.5 (136.3), 133.8, 129.6 (129.5), 129.1, 128.4 (128.3), 126.4, 125.8, 125.52 (125.47), 122.70 (122.68), 122.1 (122.0), 79.10 (79.07), 62.8 (d, *J* = 6.2 Hz) (62.7 (d, *J* = 3.8 Hz)), 62.4 (62.3), 33.5 (d, *J* = 138.8 Hz) (32.5 (d, *J* = 138.4 Hz)), 16.5 (d, *J* = 5.1 Hz) (16.4 (d, *J* = 4.9 Hz)), 12.7 (d, *J* = 6.0 Hz) (12.6 (d, *J* = 6.3 Hz)). ^{31}P NMR (162 MHz, $CDCl_3$) δ 24.94 (24.87). HRMS-ESI (*m/z*): calcd for $C_{19}H_{25}NO_4P^+$ $[M+H]^+$: 362.1516, found 362.1521.

Copies of ^{1}H, ^{13}C, and ^{31}P NMR spectra of products **3**

^{1}H and ^{13}C NMR of compound **3aa**

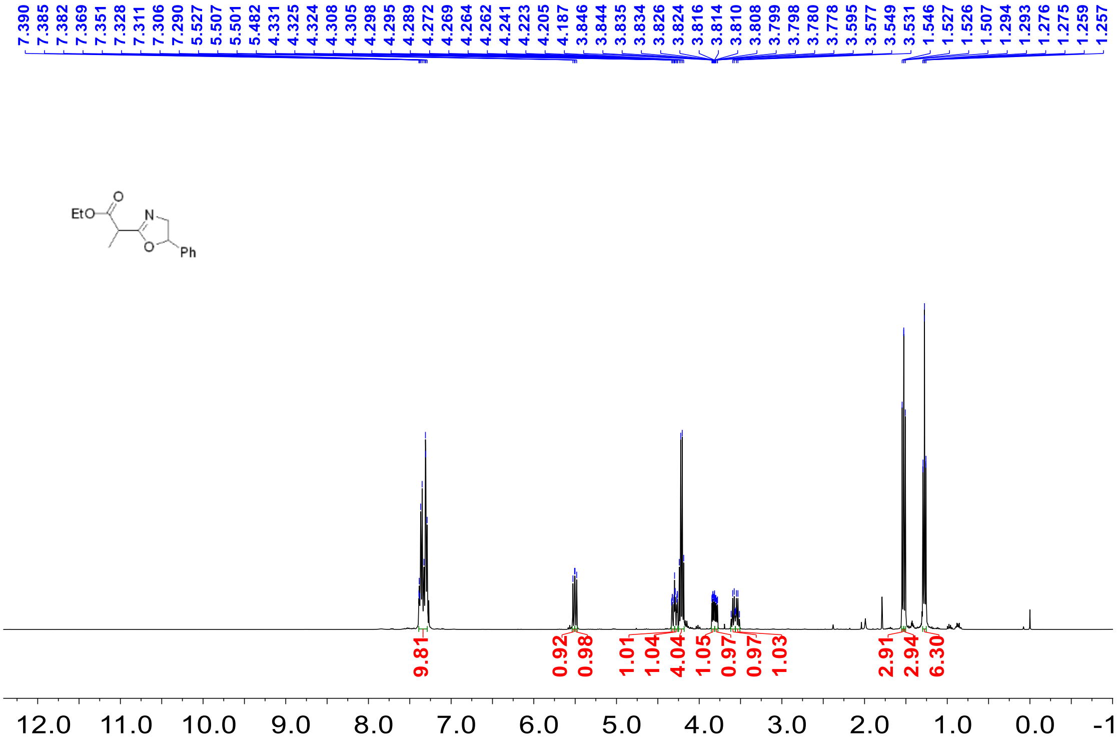

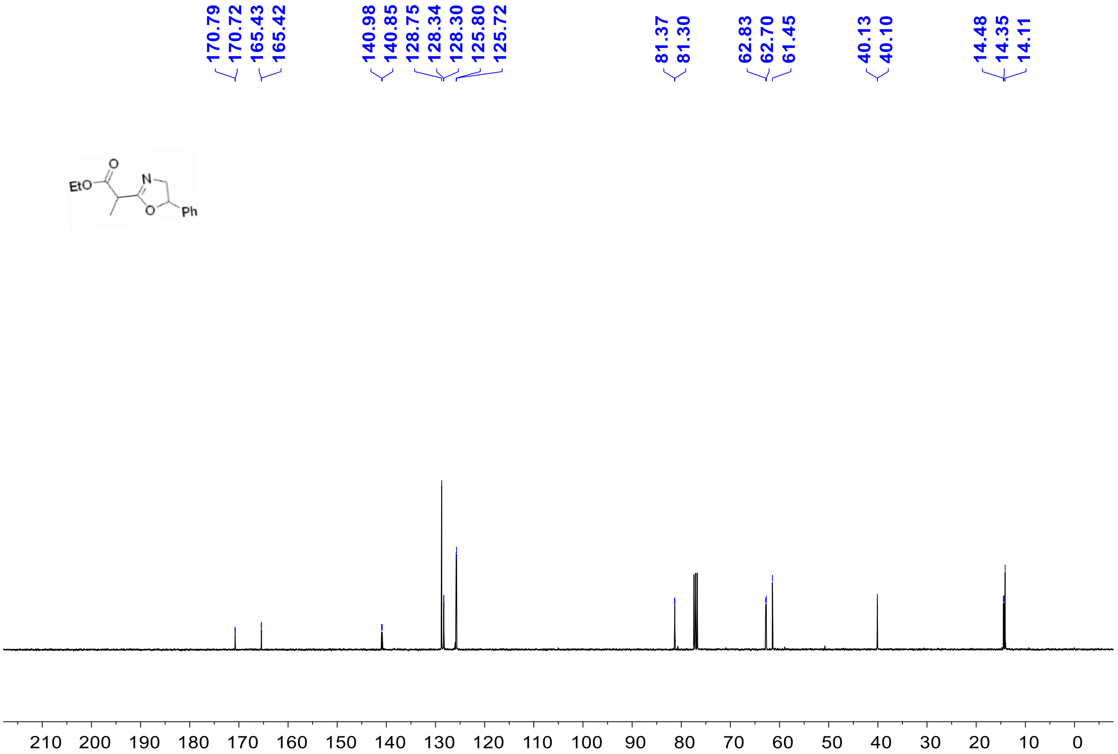

^{1}H and ^{13}C NMR of compound **3ab**

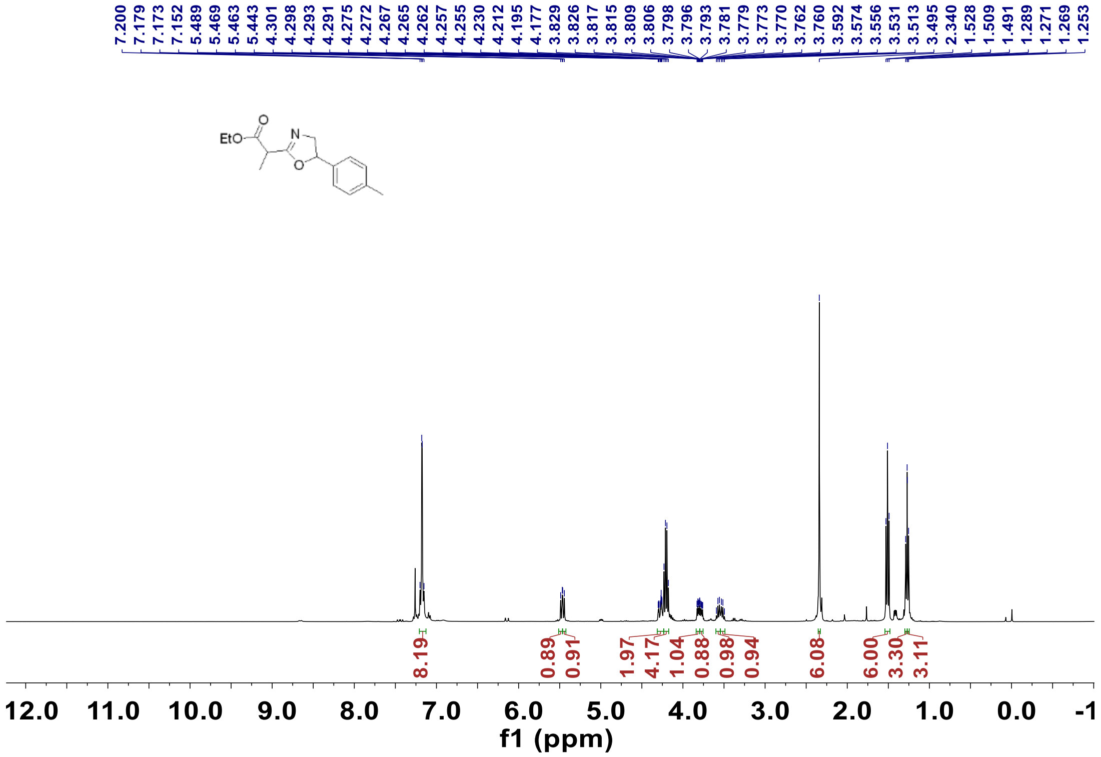

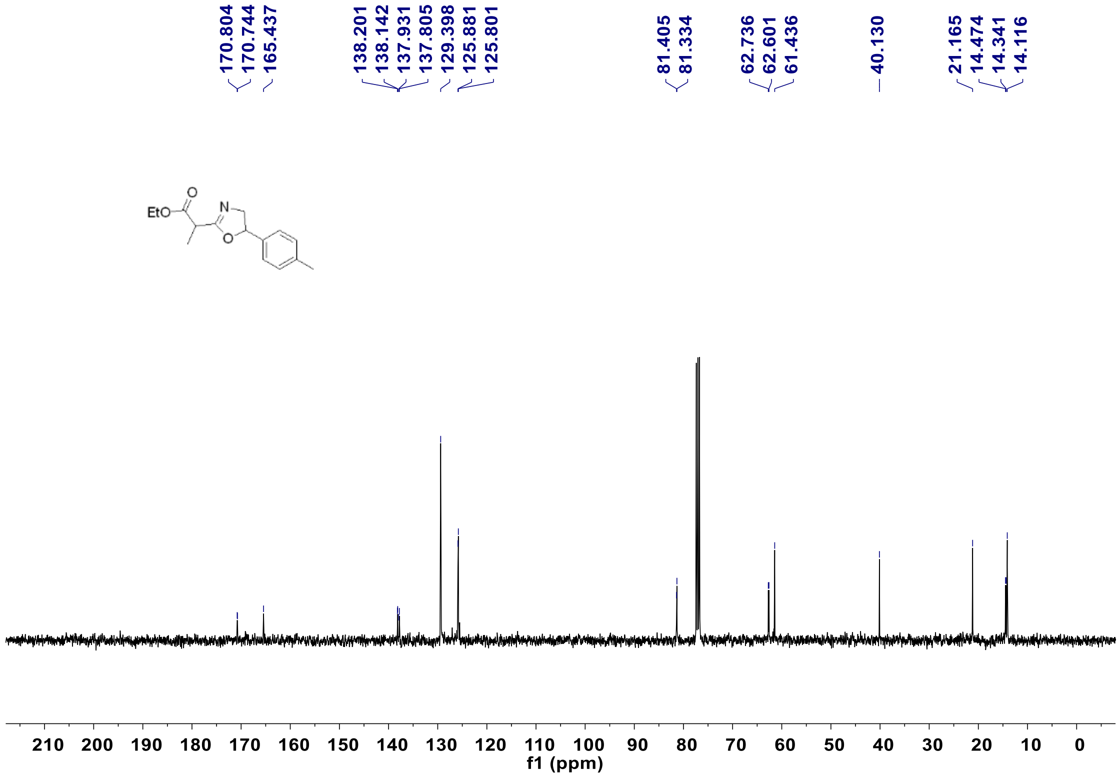

^{1}H and ^{13}C NMR of compound **3ac**

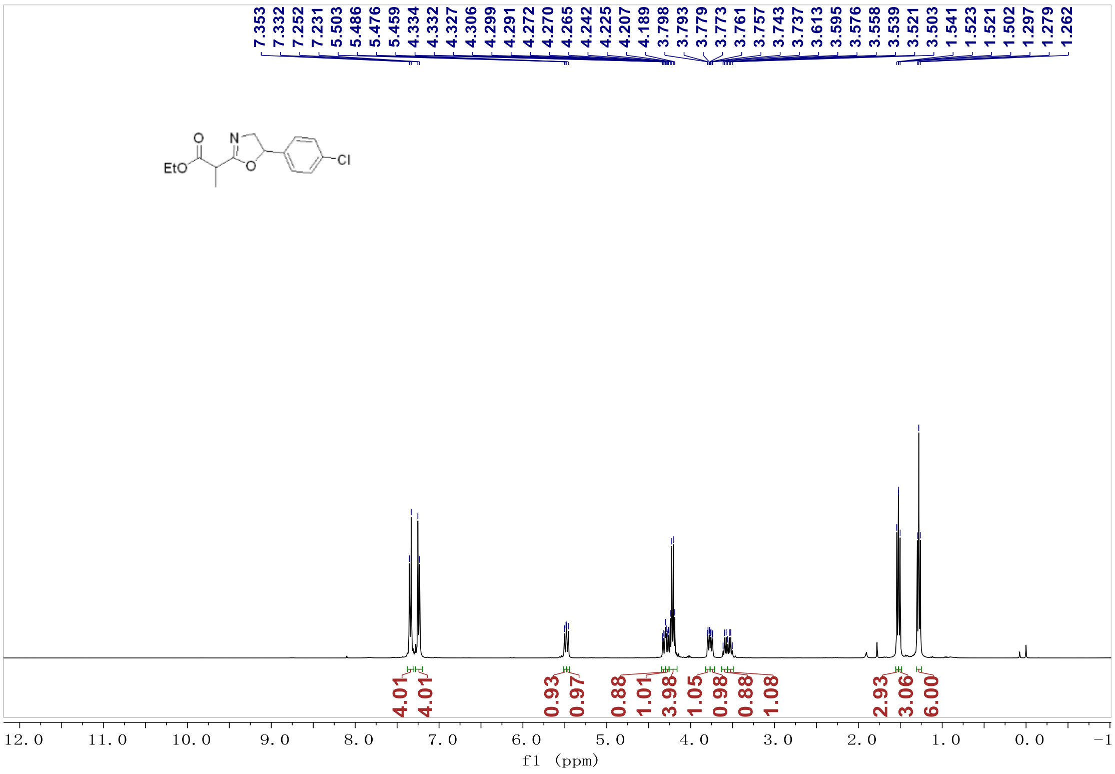

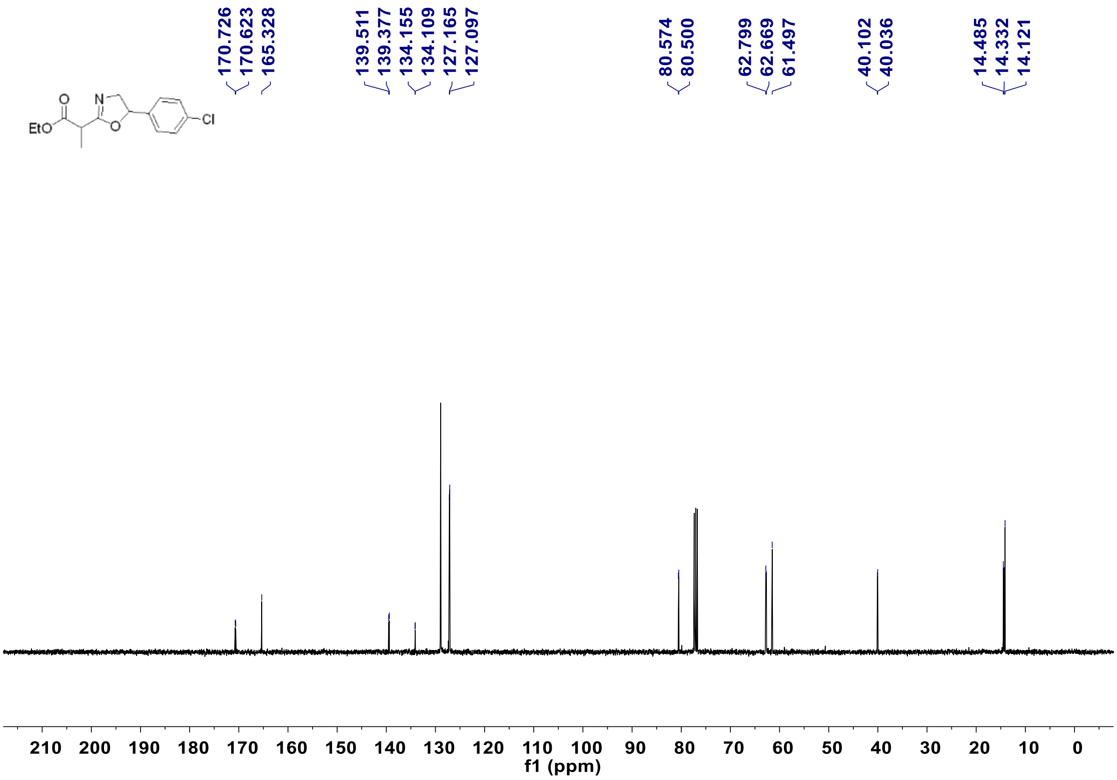

^{1}H and ^{13}C NMR of compound **3ad**

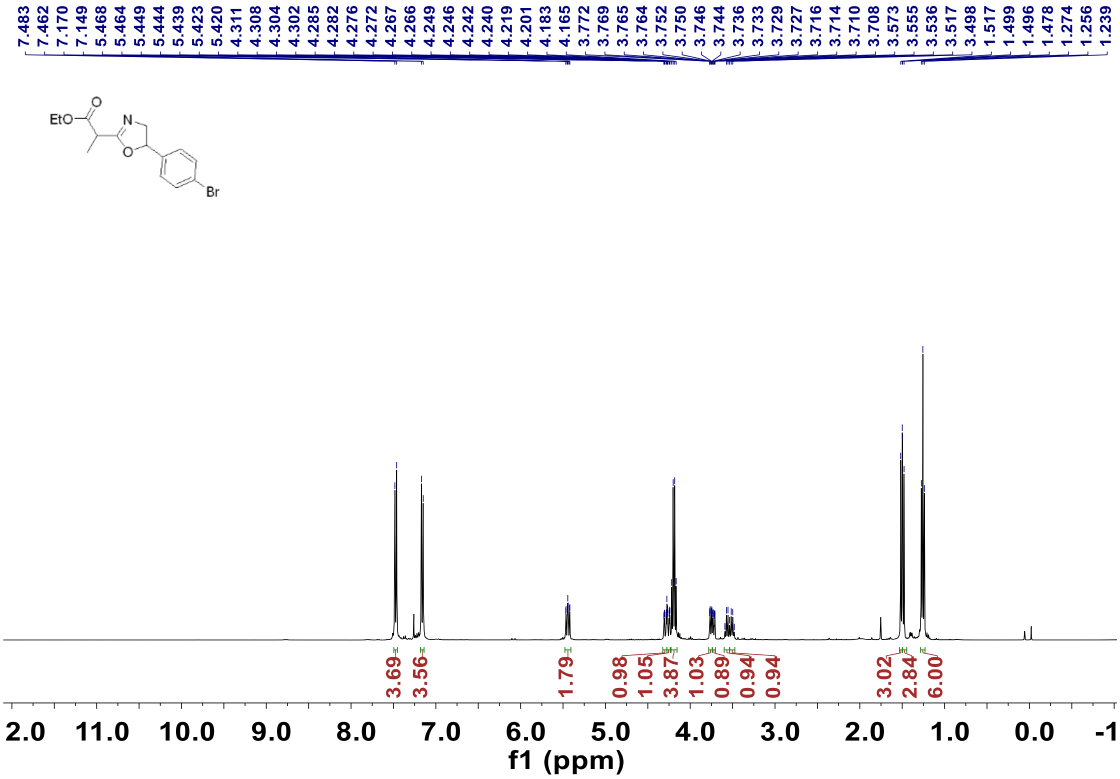

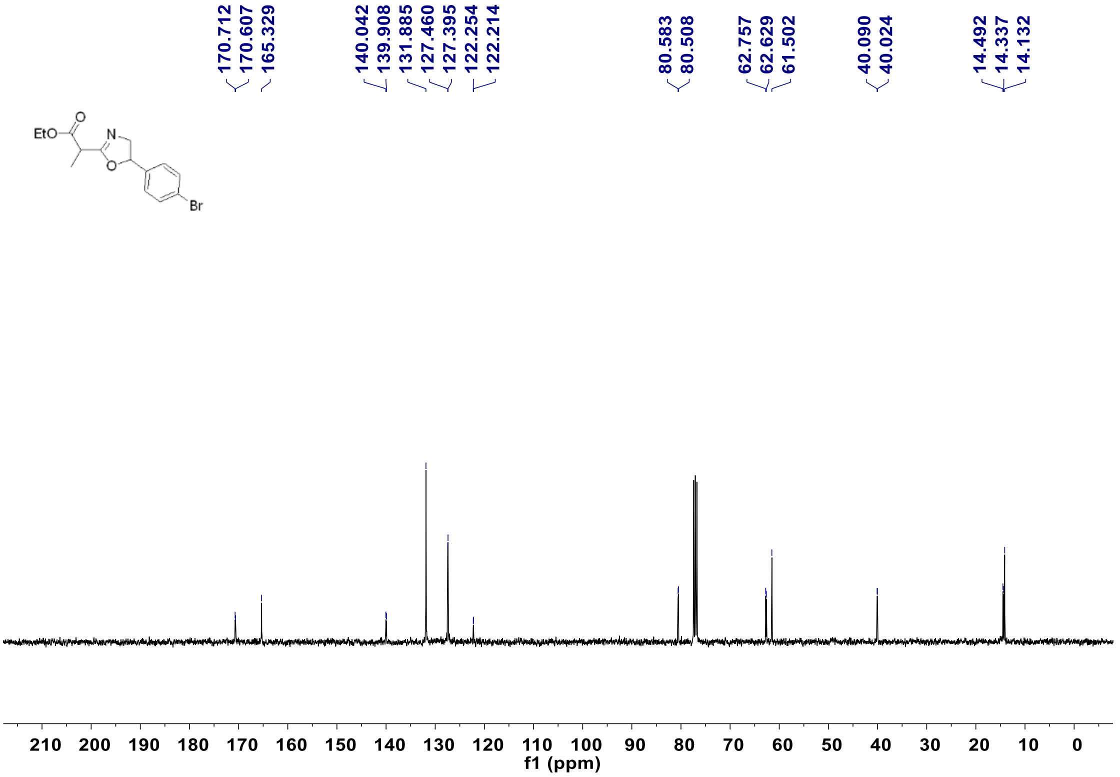

^{1}H and ^{13}C NMR of compound **3ae**

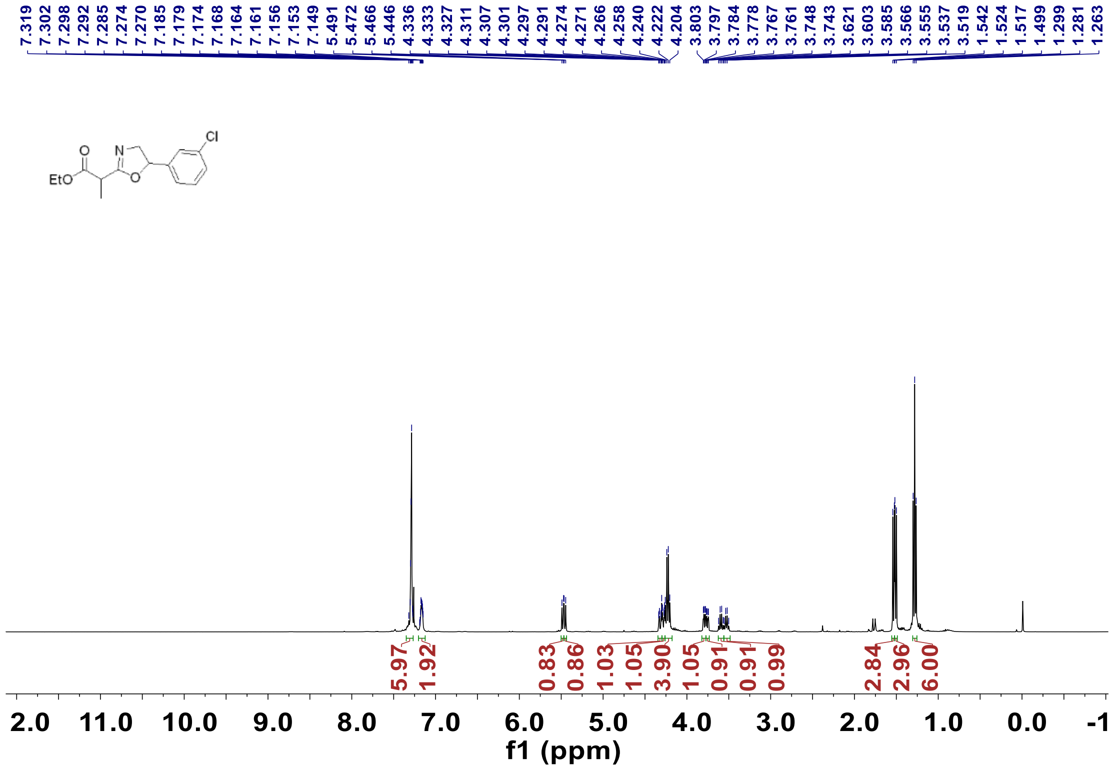

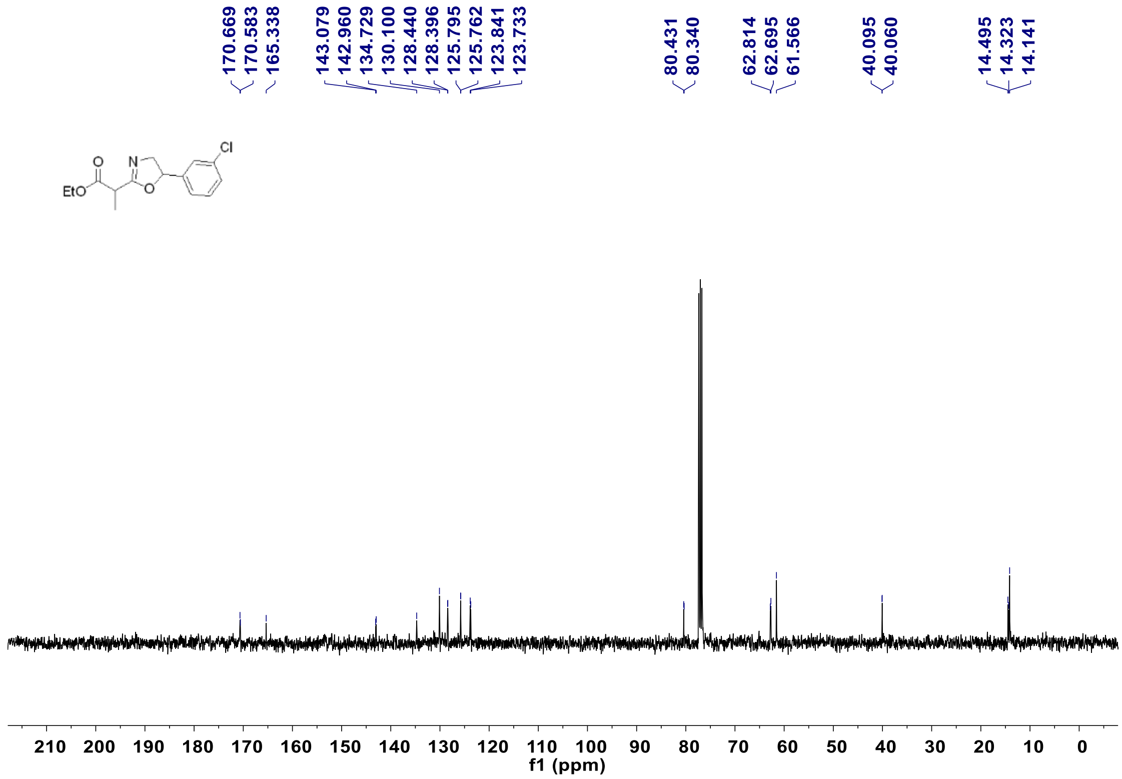

^{1}H and ^{13}C NMR of compound **3af**

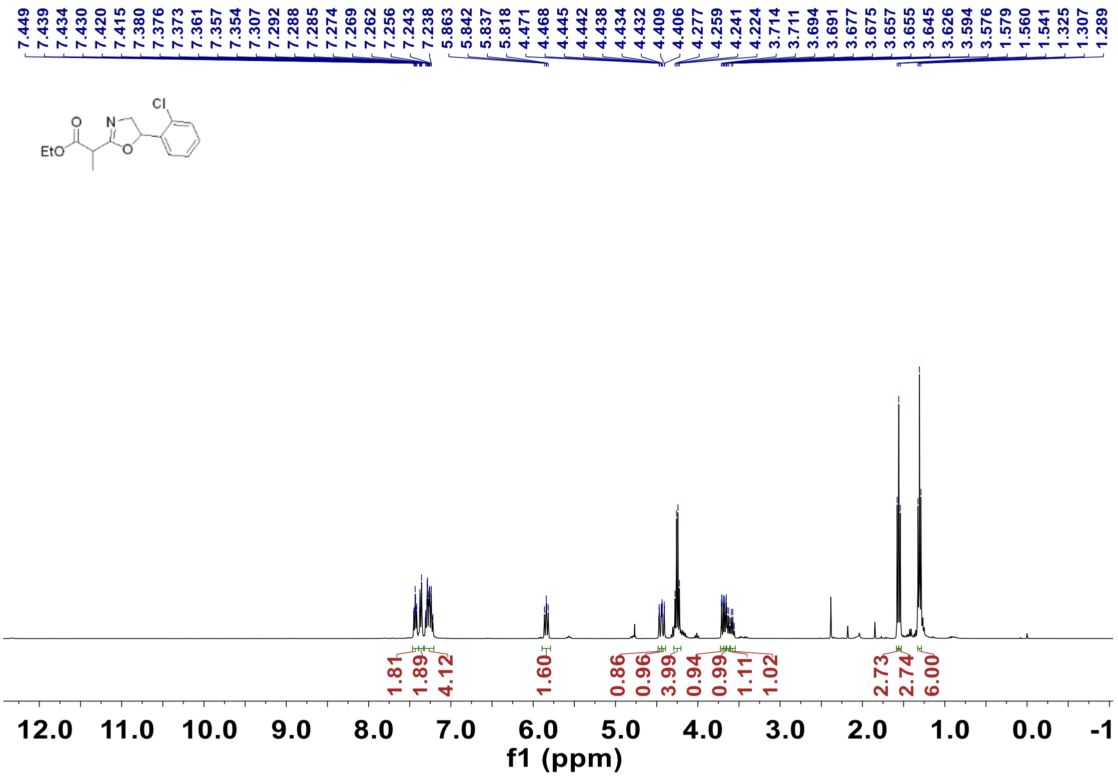

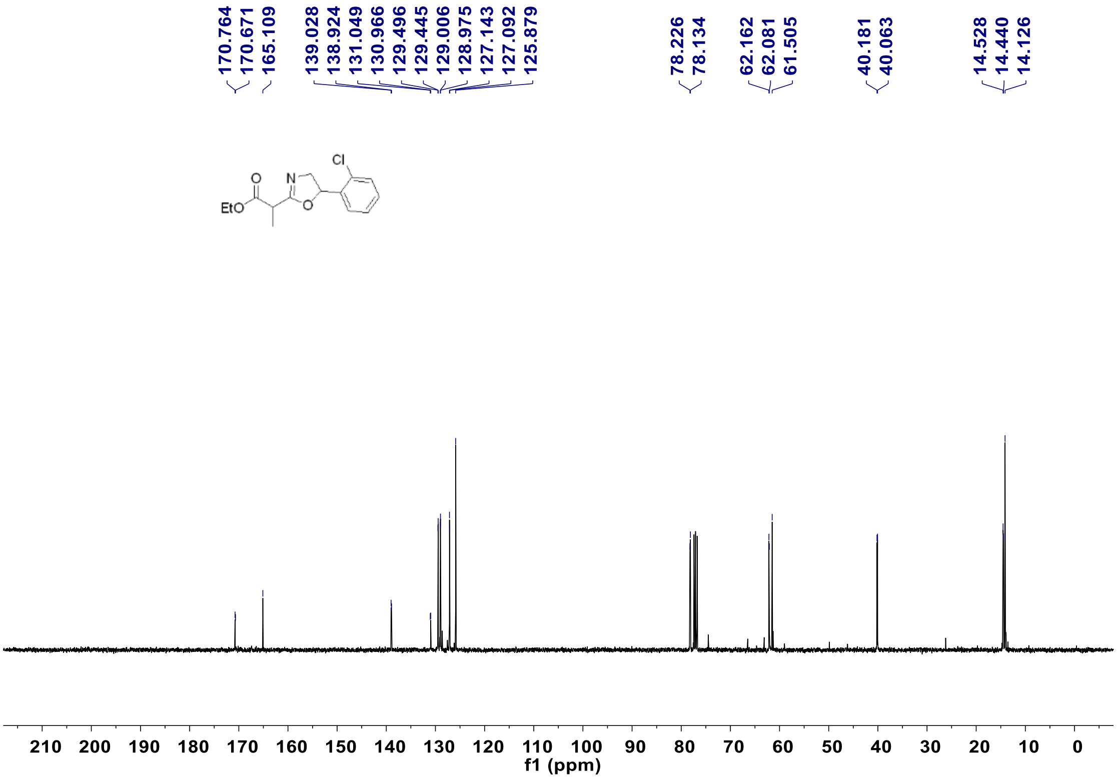

^{1}H and ^{13}C NMR of compound **3ag**

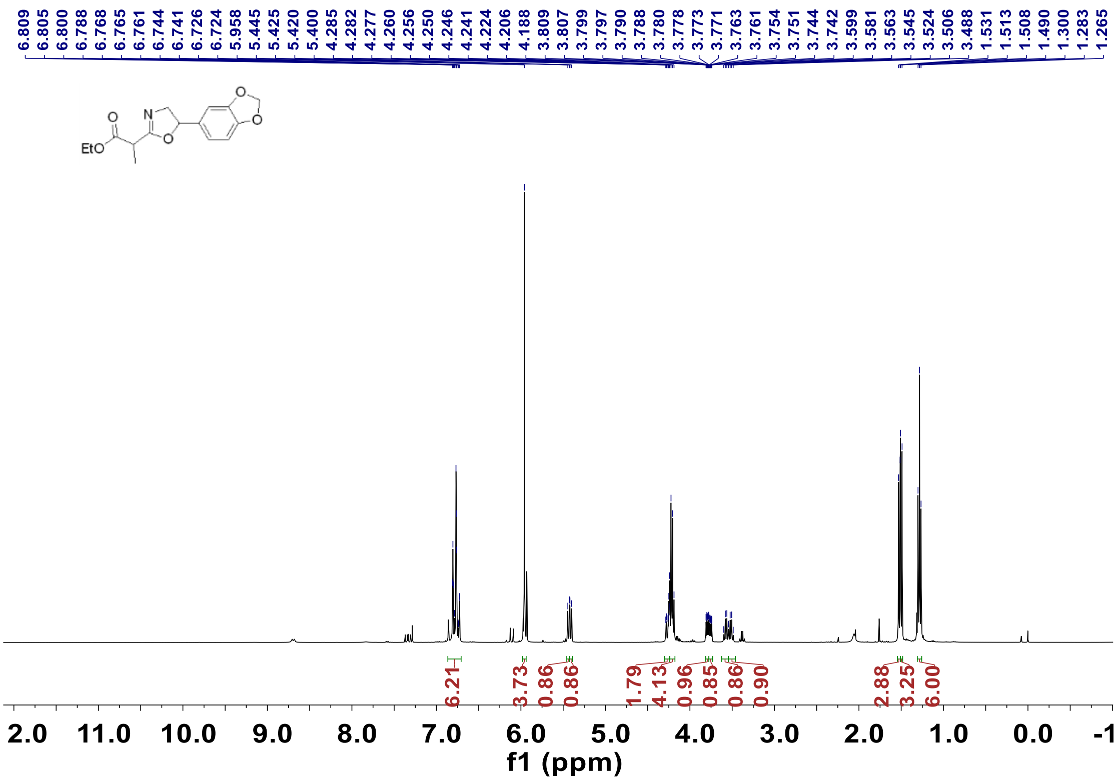

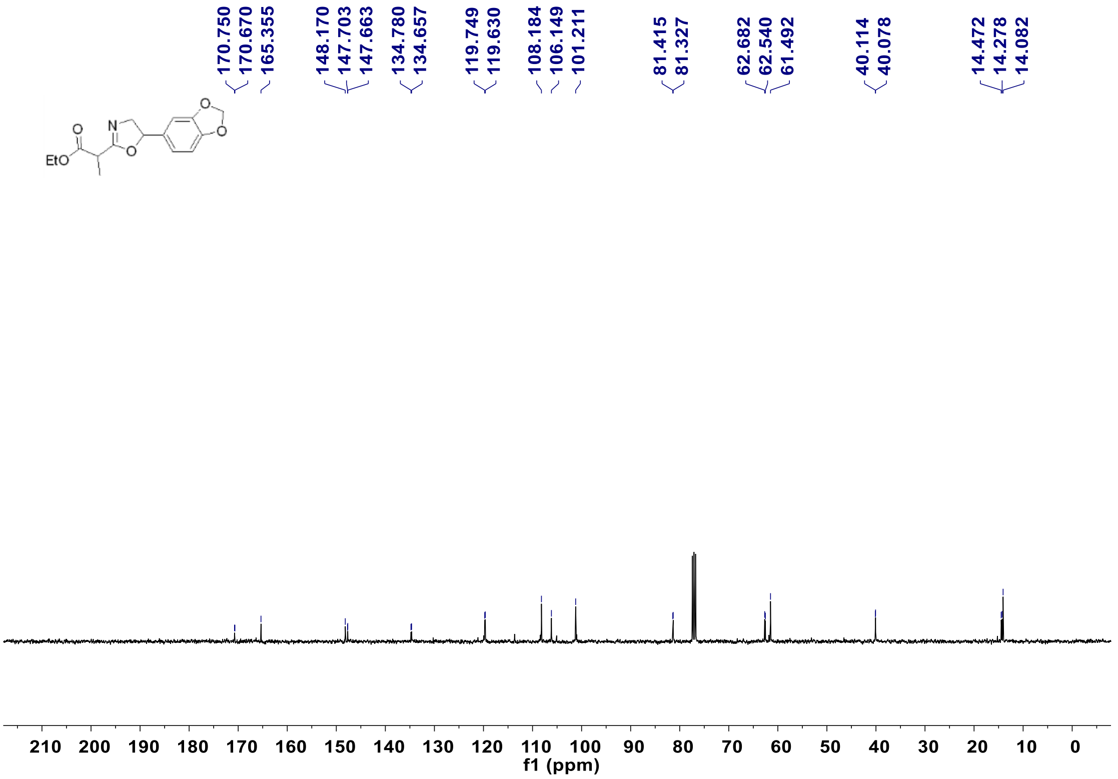

^{1}H and ^{13}C NMR of compound **3ah**

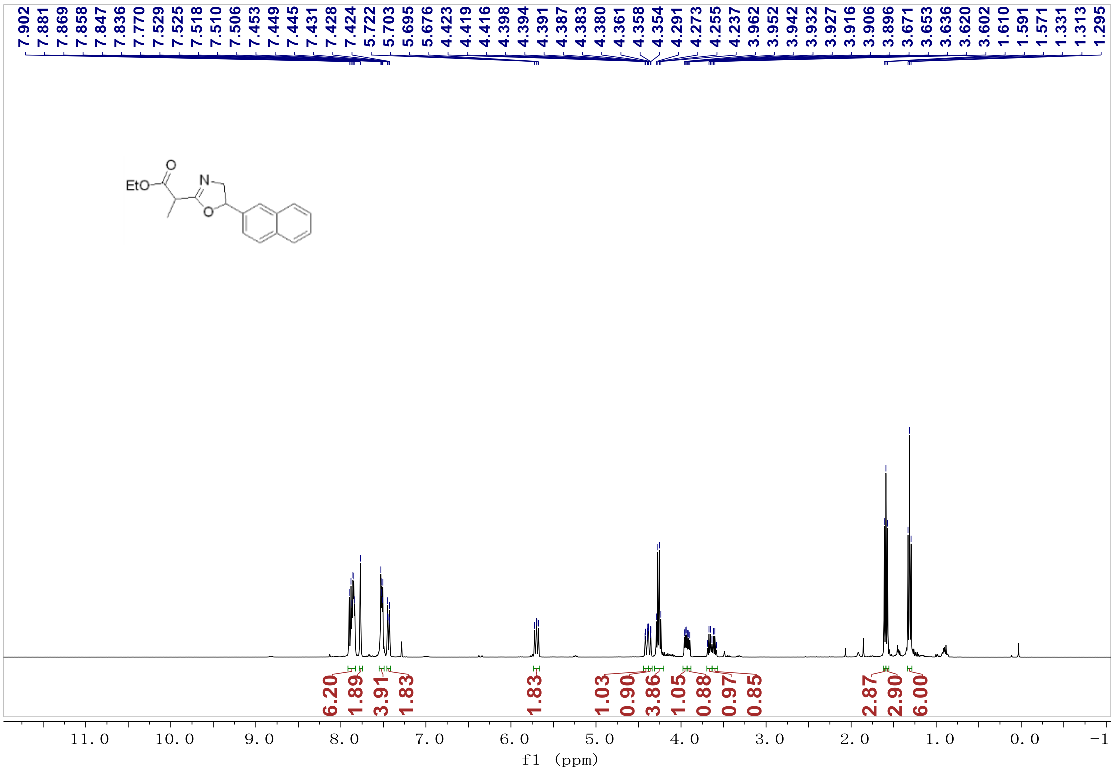

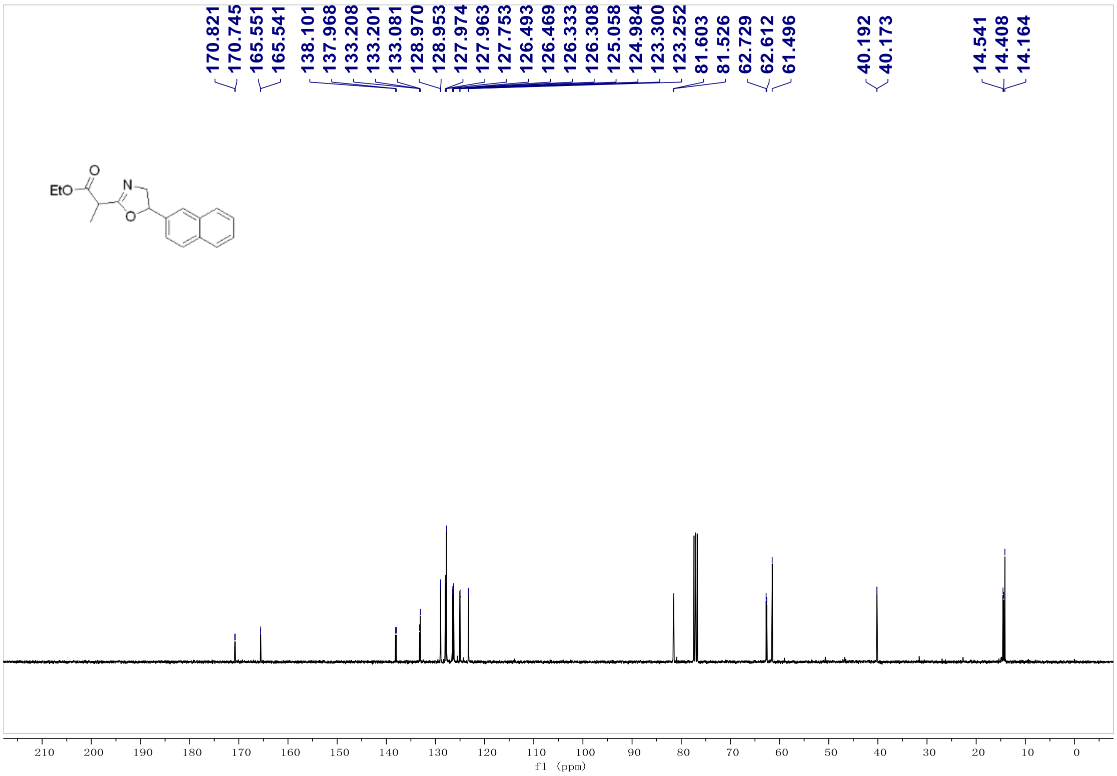

^{1}H and ^{13}C NMR of compound **3ai**

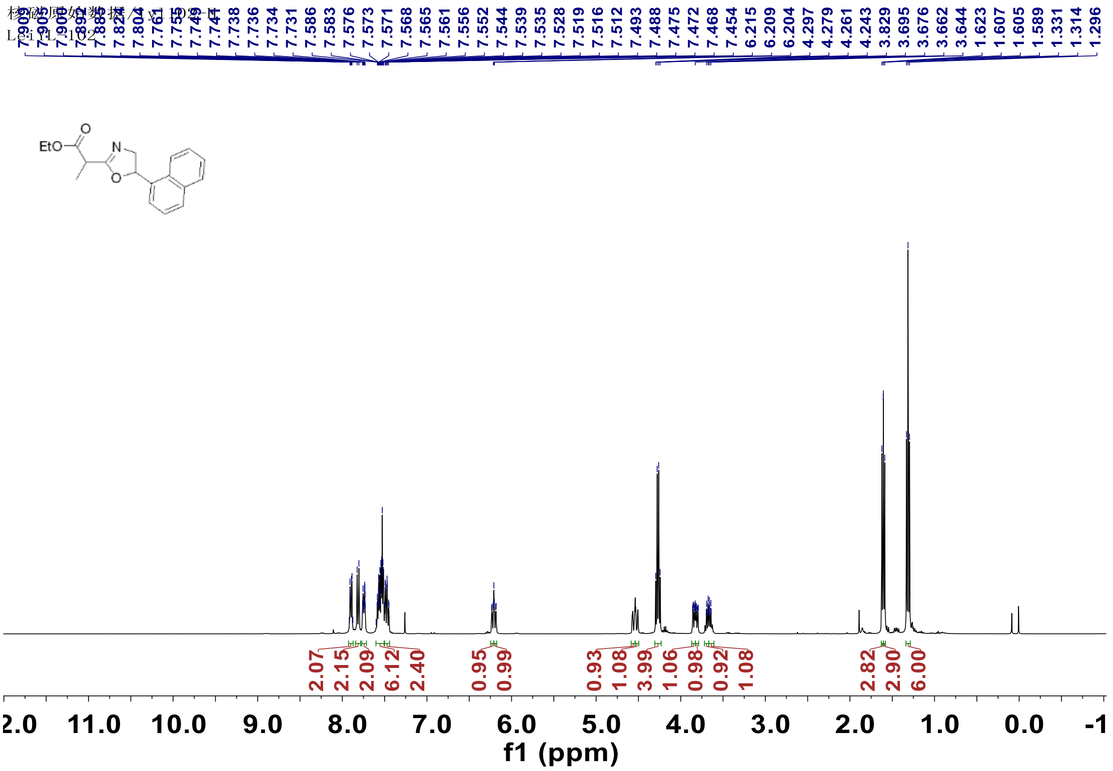

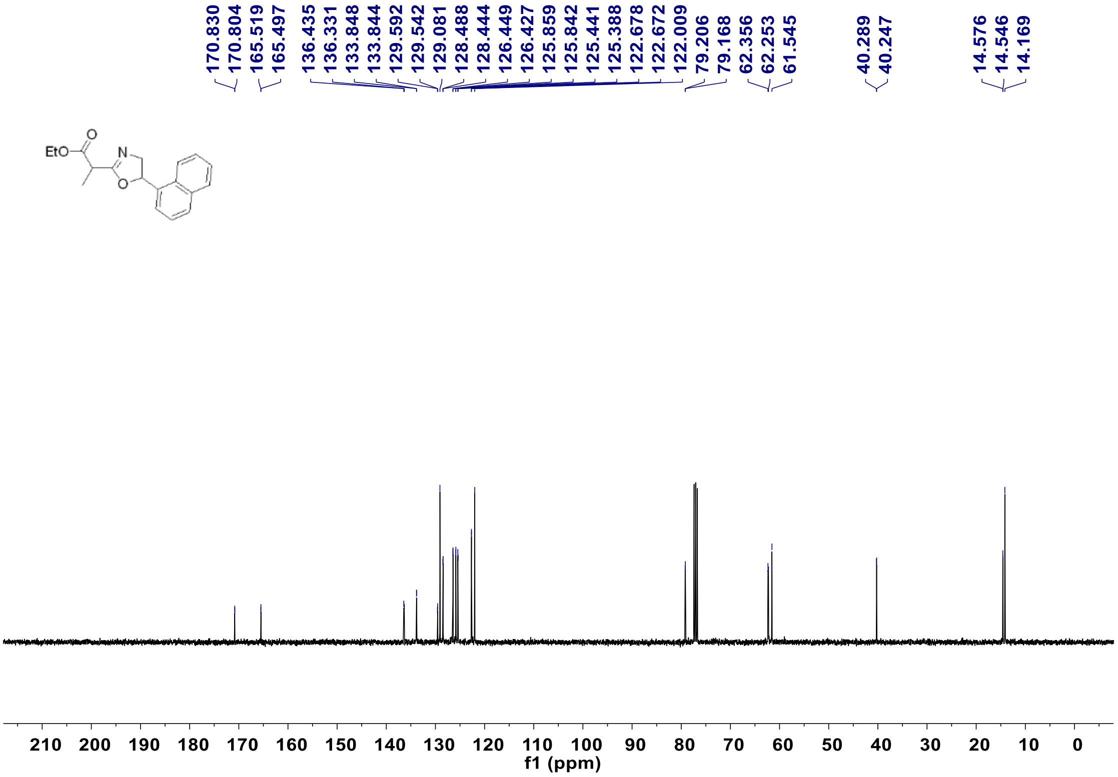

^{1}H and ^{13}C NMR of compound **3bi**

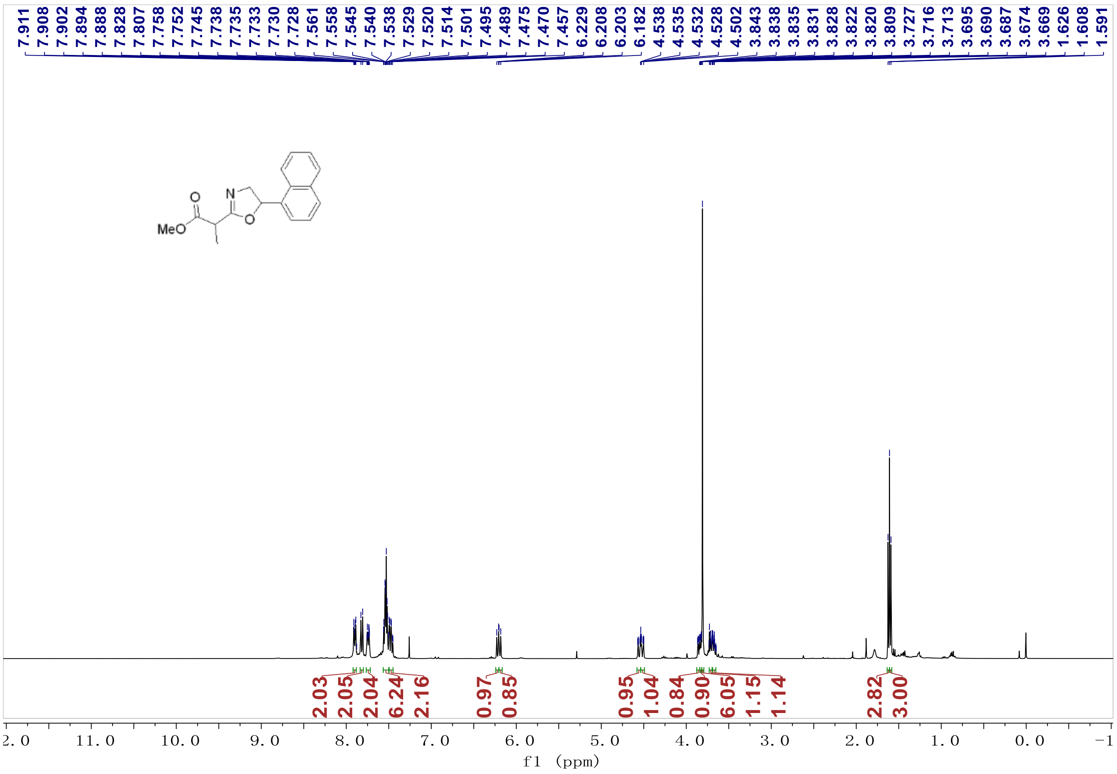

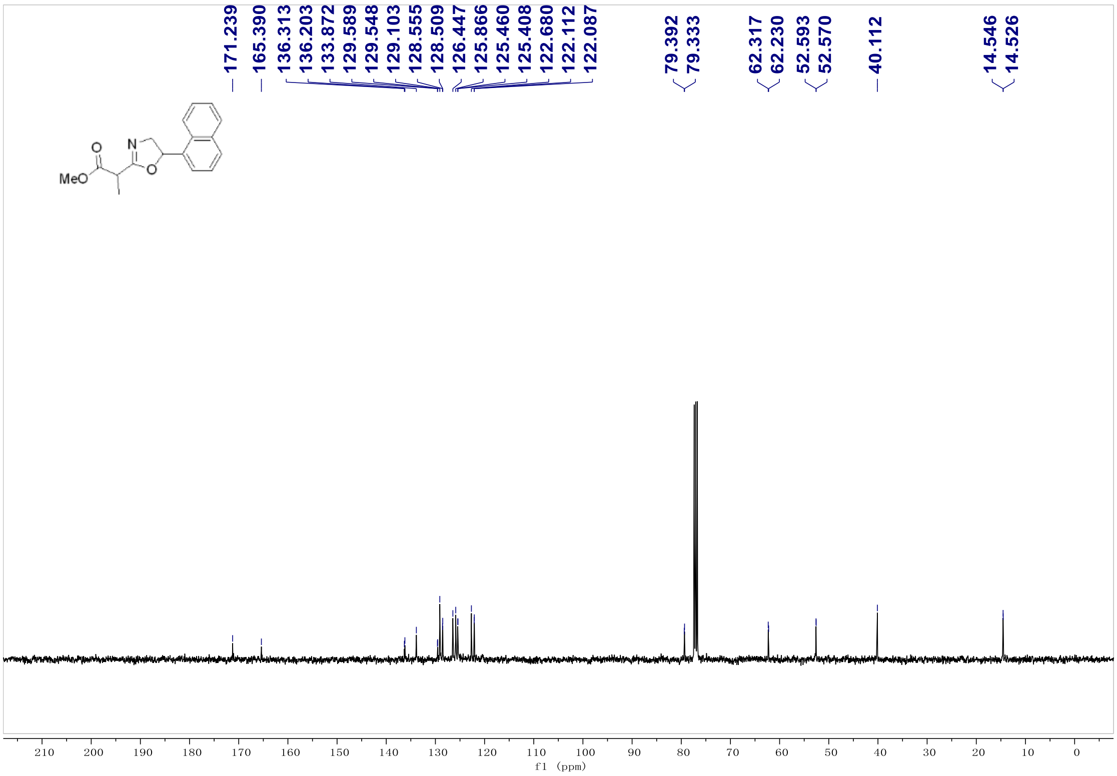

^{1}H and ^{13}C NMR of compound **3ci**

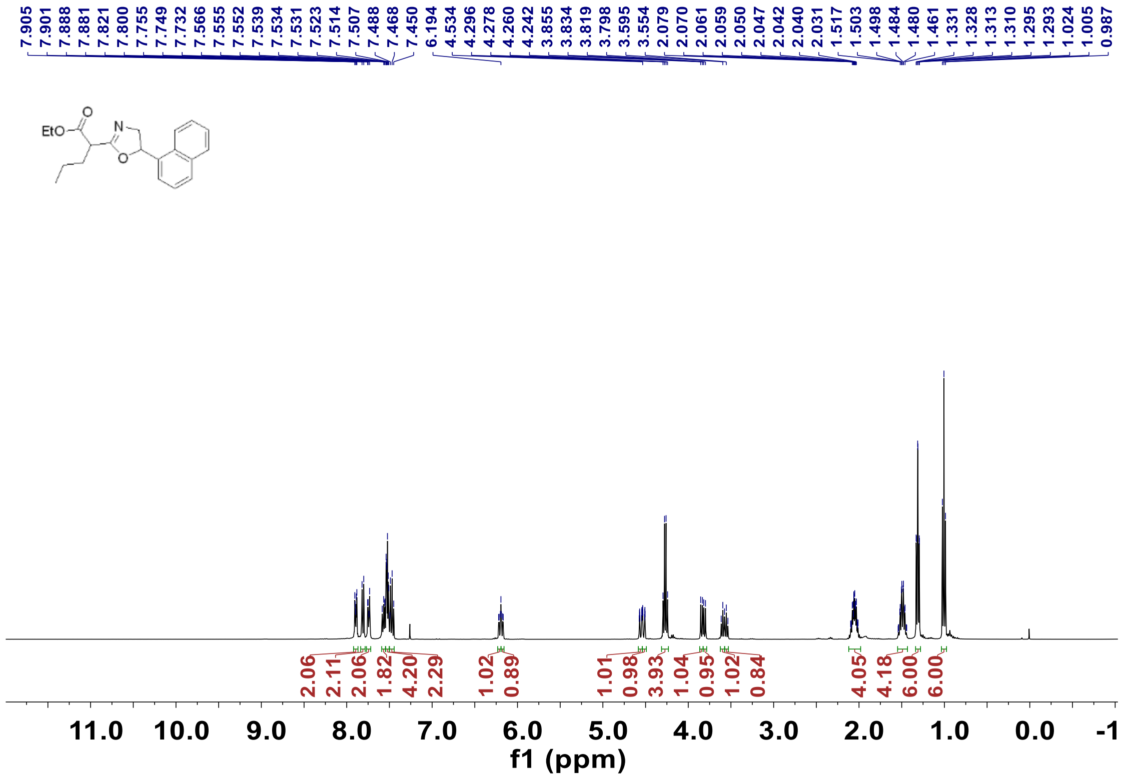

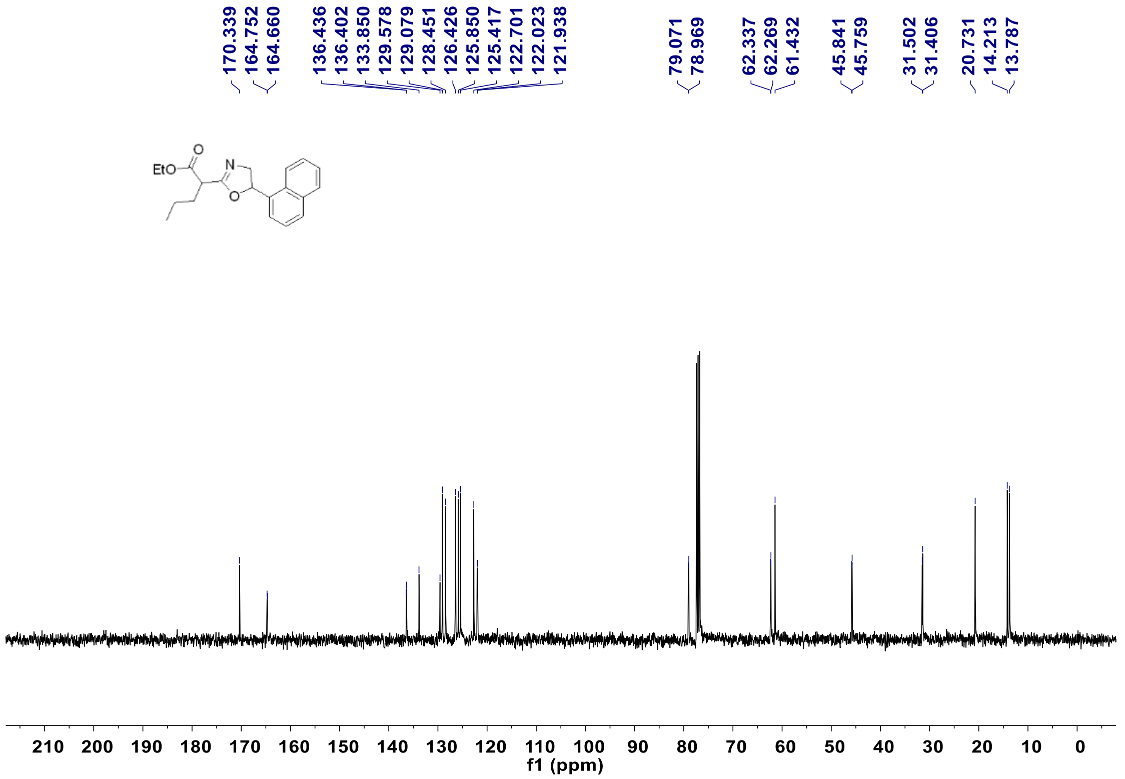

^{1}H and ^{13}C NMR of compound **3di**

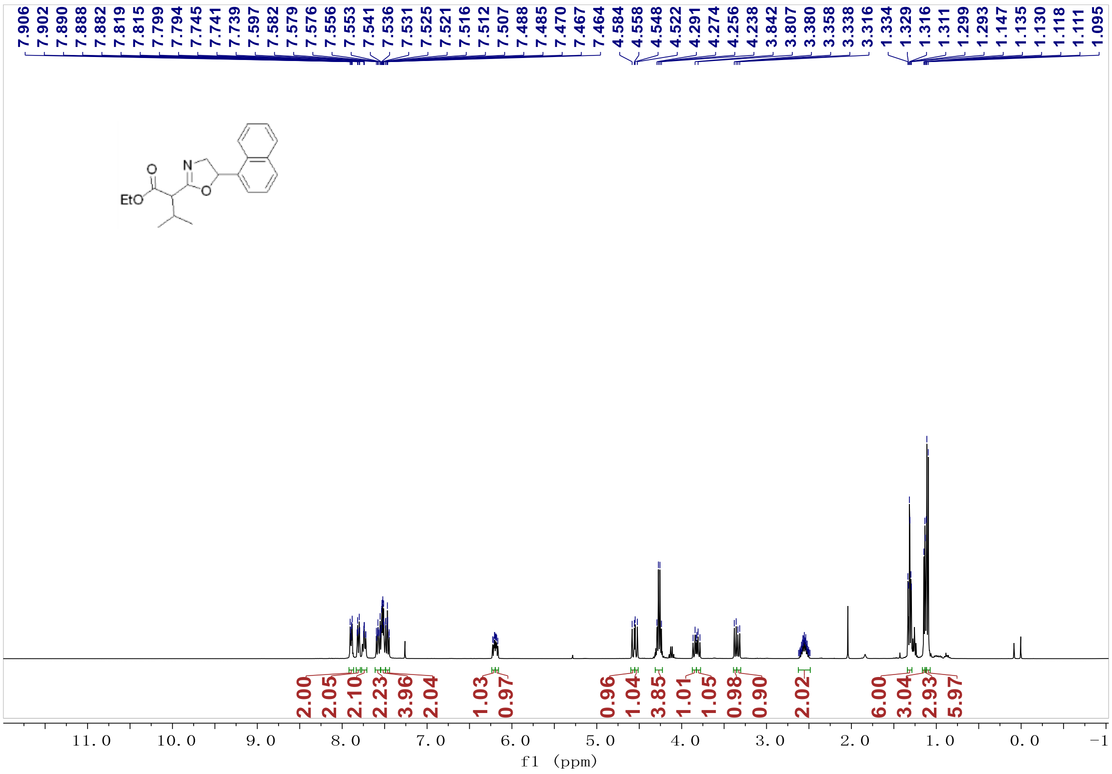

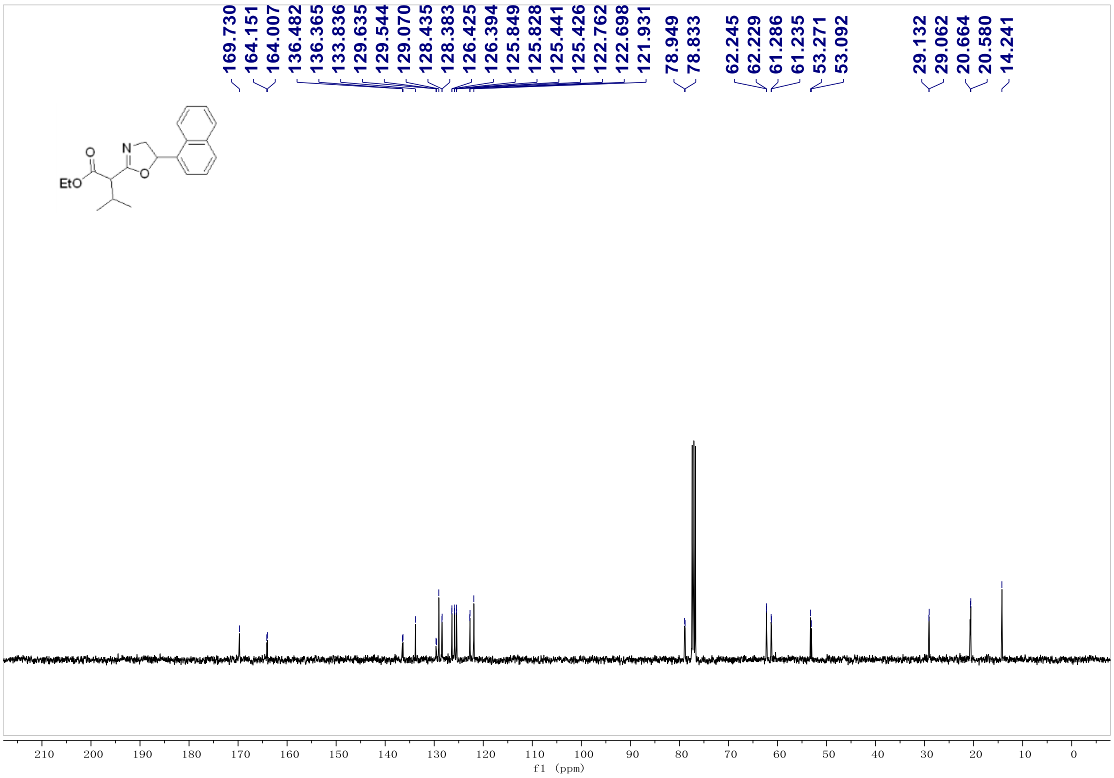

^{1}H and ^{13}C NMR of compound **3ei**

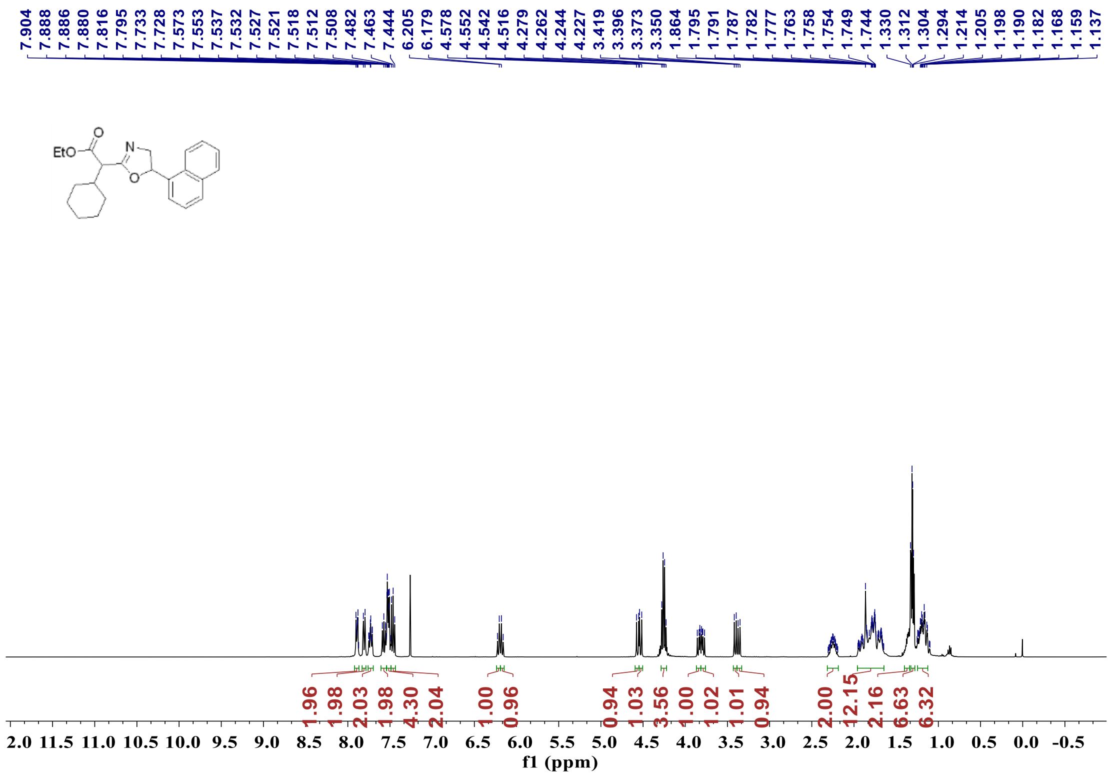

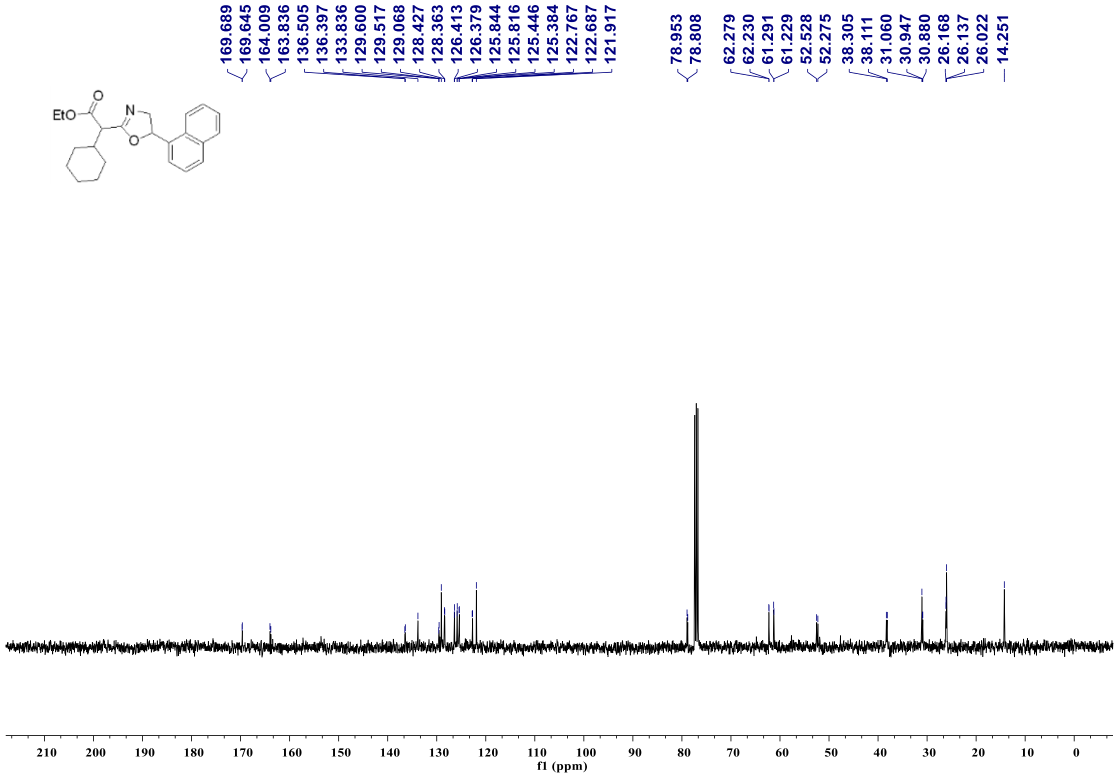

^{1}H and ^{13}C NMR of compound **3fi**

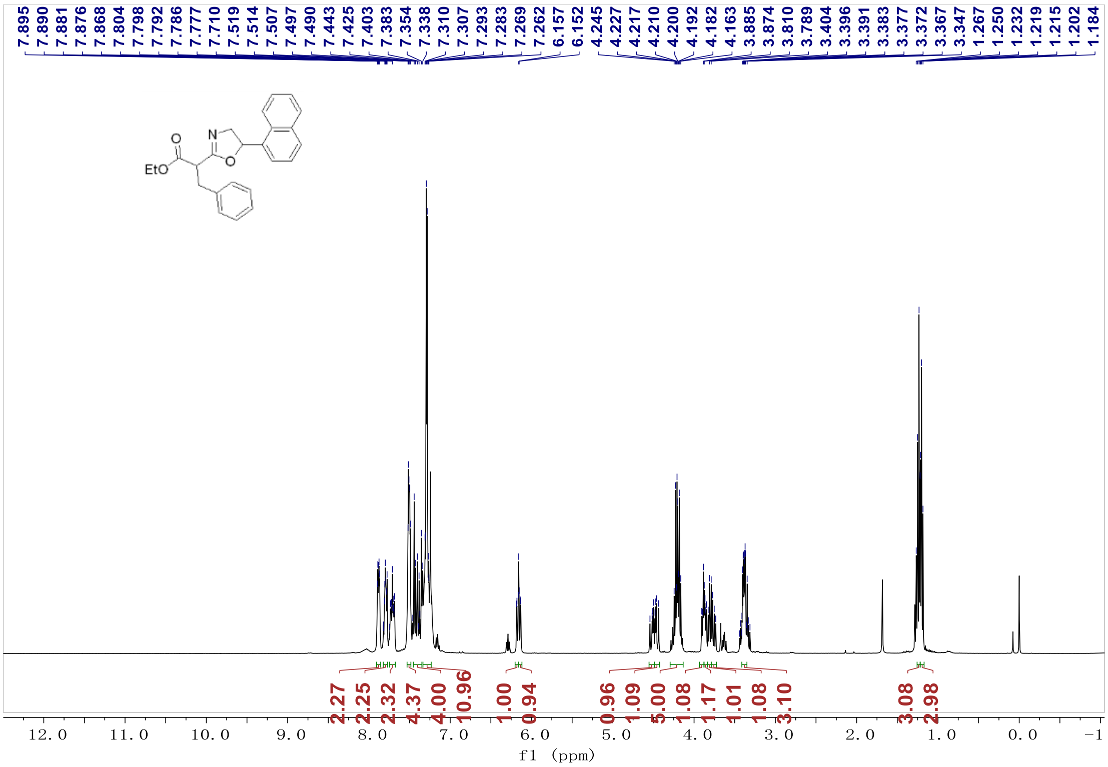

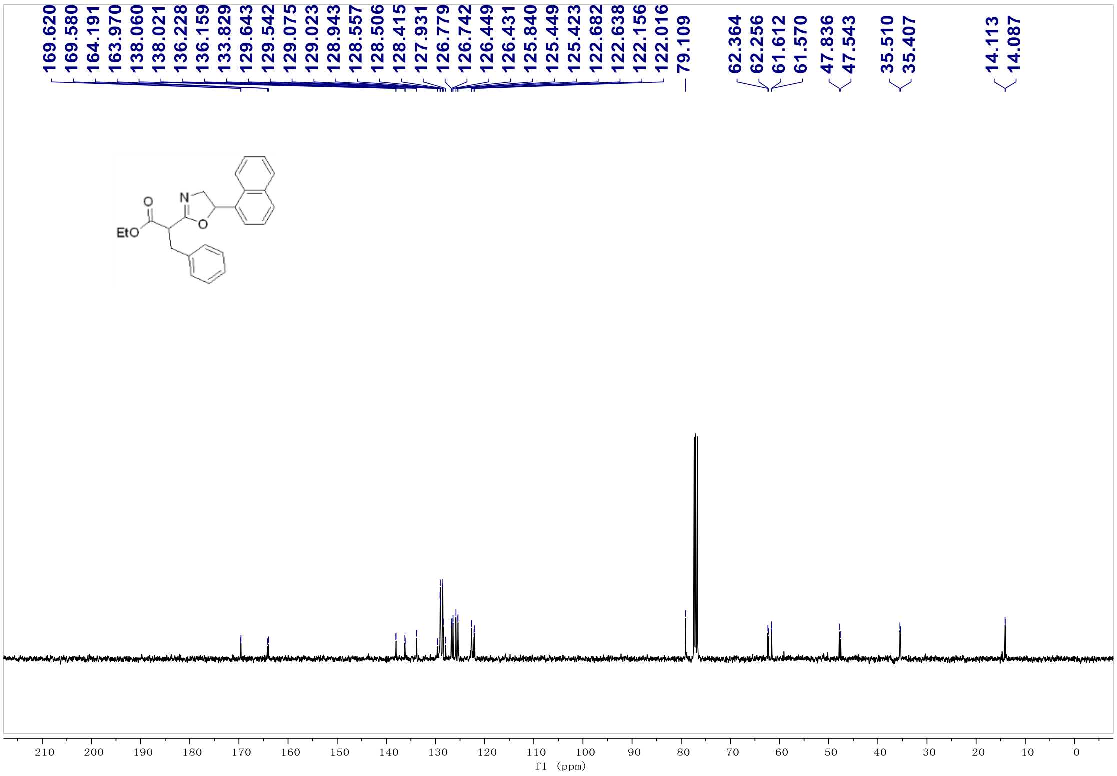

^{1}H and ^{13}C NMR of compound **3gi**

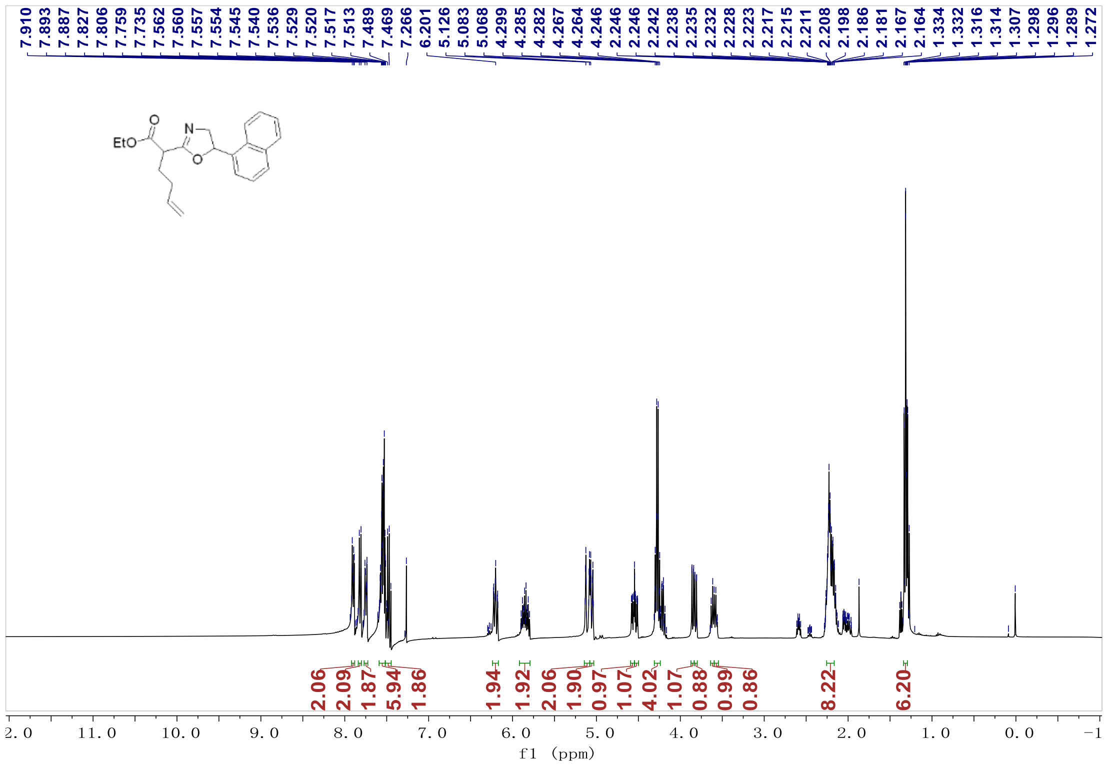

1

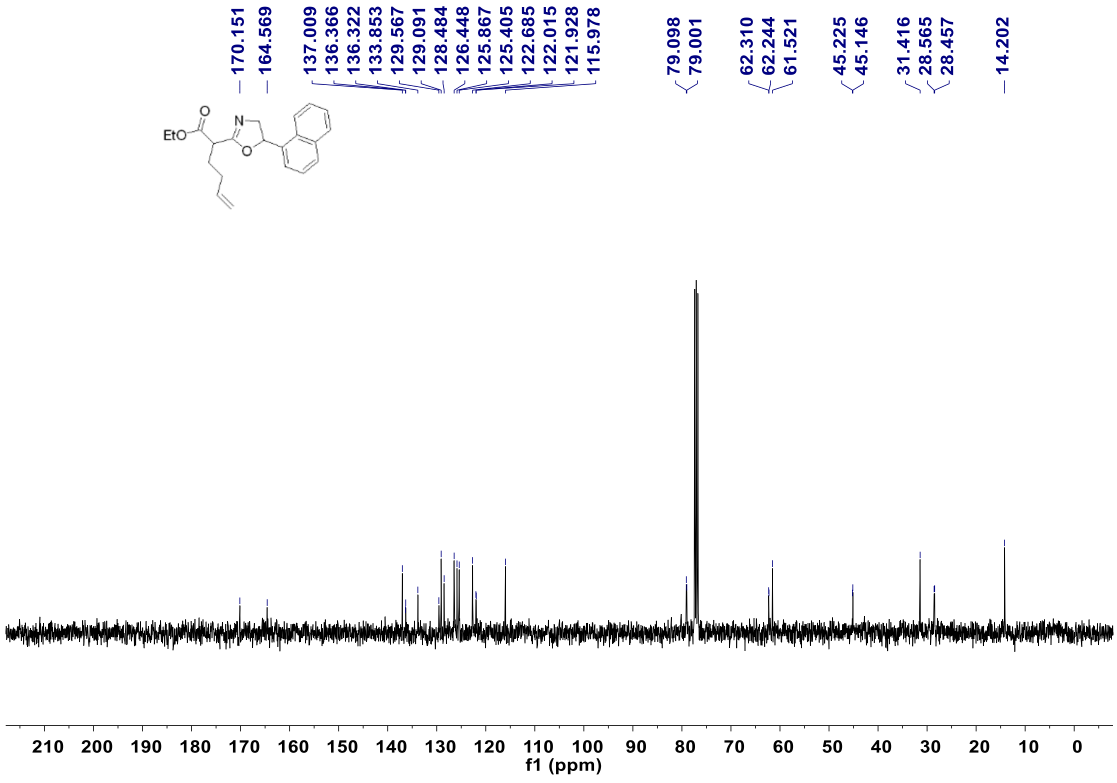

^{1}H and ^{13}C NMR of compound **3hi**

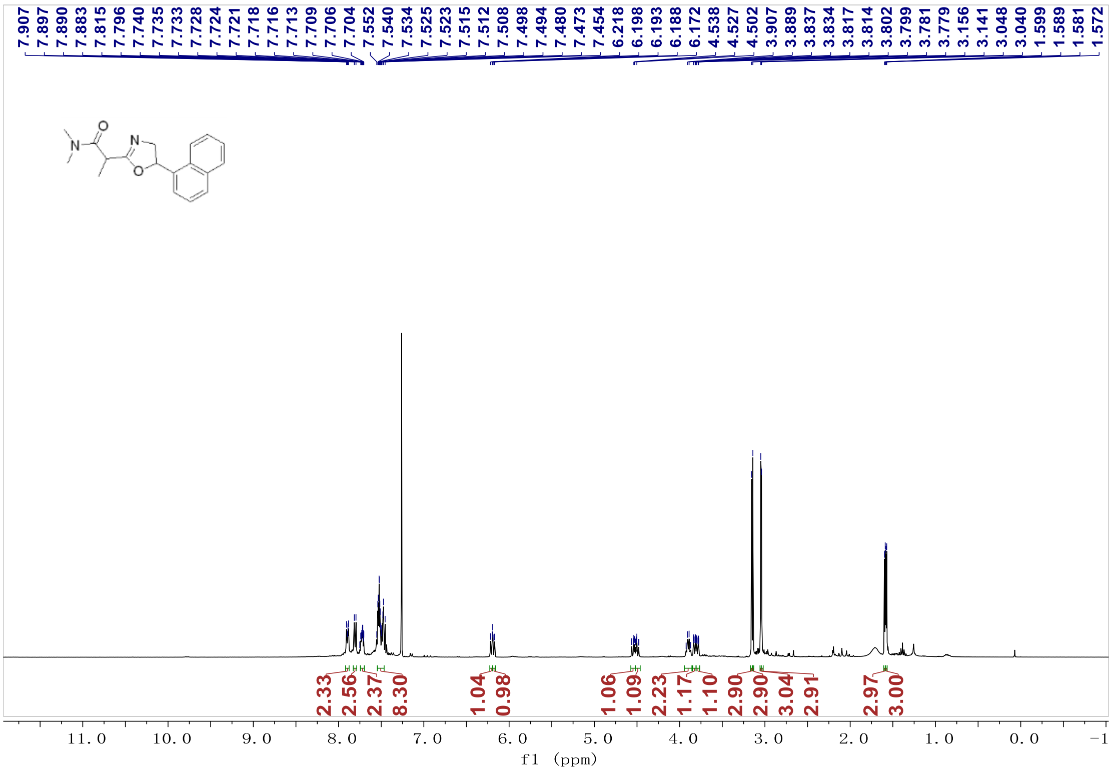

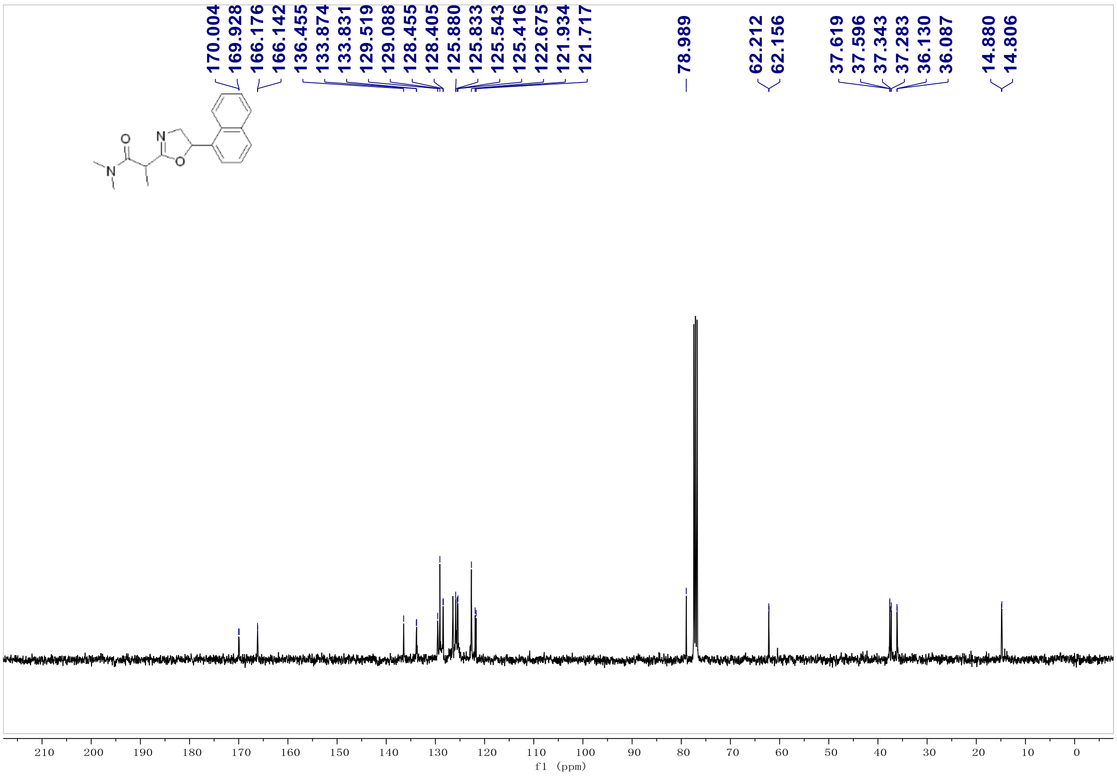

^{1}H, ^{31}P, and ^{13}C NMR of compound **3ii**

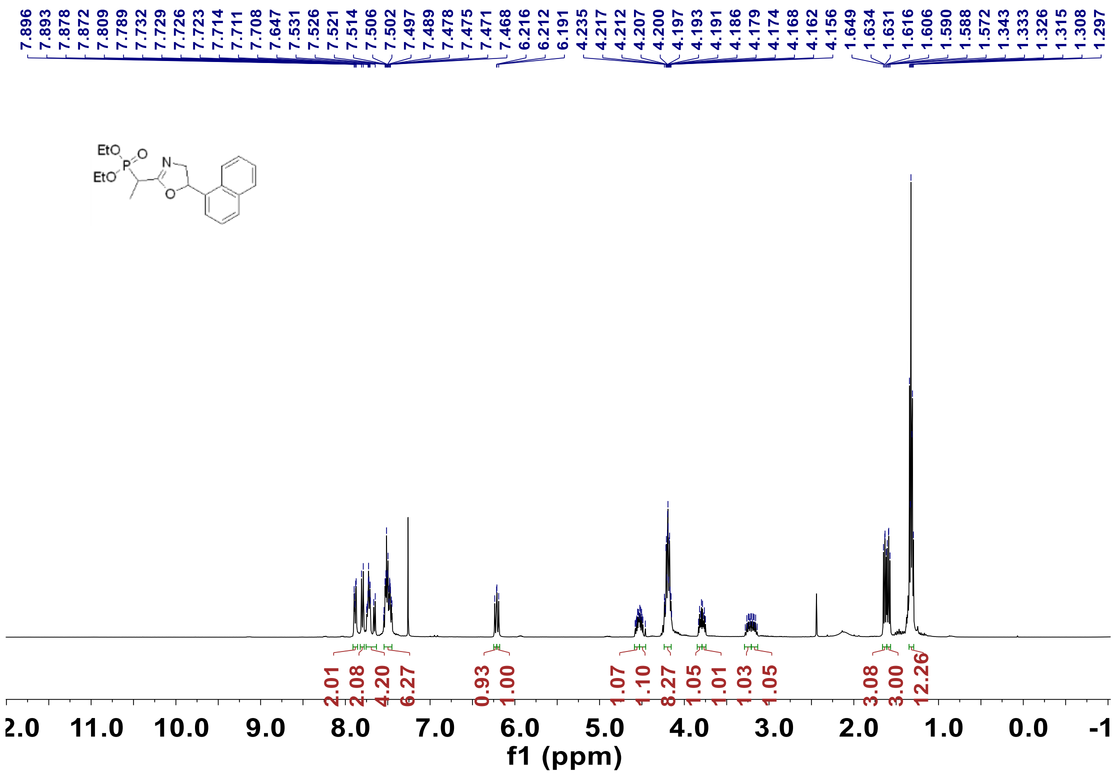

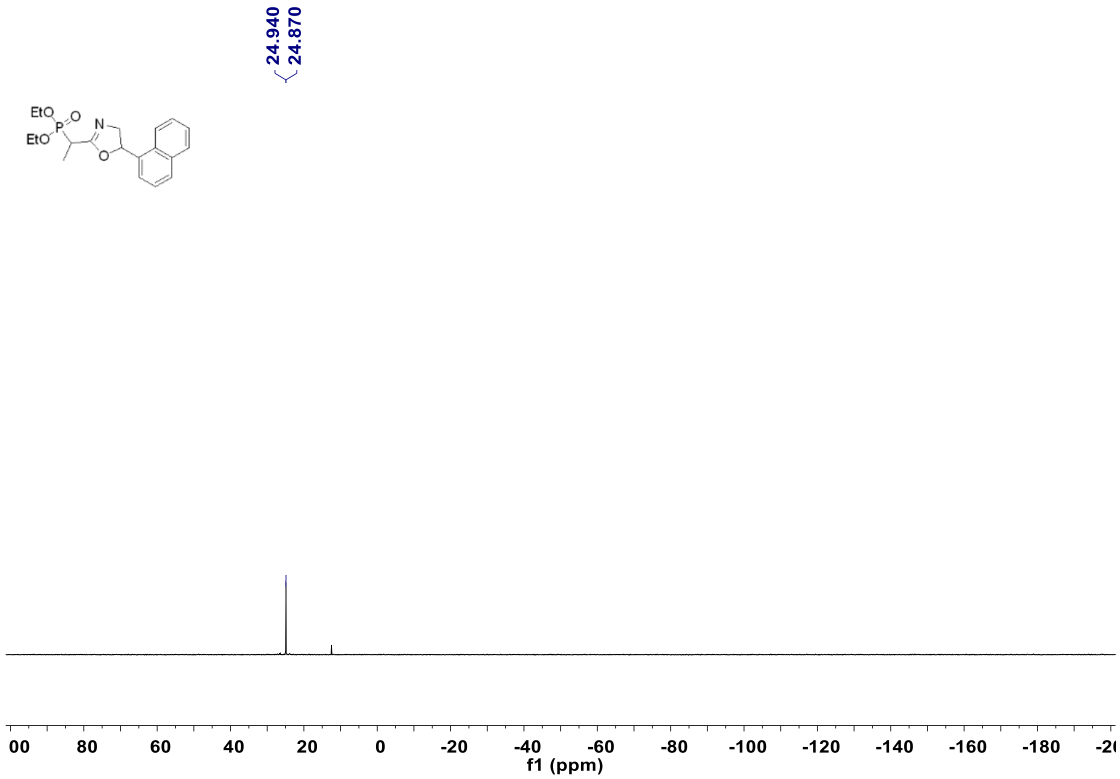

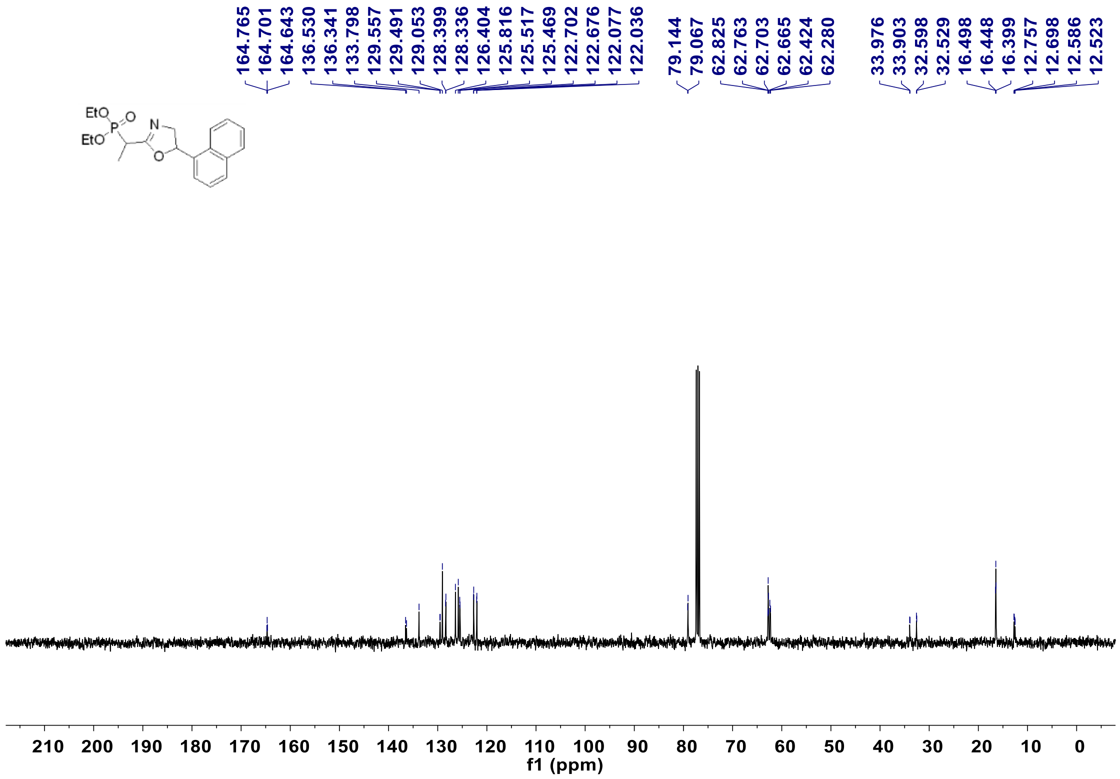
164.765
164.701
164.643
136.530
136.341
133.798
129.557
129.491
129.053
128.399
128.336
126.404
125.816
125.517
125.469
122.702
122.676
122.077
122.036
79.144
79.067
62.825
62.763
62.703
62.665
62.424
62.280
33.976
33.903
32.598
32.529
16.498
16.448
16.399
12.757
12.698
12.586
12.523
EtO
EtO
P
O
N
O
210 200 190 180 170 160 150 140 130 120 110 100 90 80 70 60 50 40 30 20 10 0
f1 (ppm)
